# Fish Capsules: A System for High‐Throughput Screening of Combinatorial Drugs

**DOI:** 10.1002/advs.202104449

**Published:** 2022-01-27

**Authors:** Minghui Tang, Xin Duan, Anqi Yang, Shijie He, Yajing Zhou, Yuxin Liu, Lu Zhang, Xuan Luo, Peng Shi, Honglin Li, Xudong Lin

**Affiliations:** ^1^ Guangdong Provincial Key Laboratory of Sensor Technology and Biomedical Instrument School of Biomedical Engineering Sun Yat‐Sen University Guangzhou 510006 China; ^2^ Department of Biomedical Engineering City University of Hong Kong Kowloon Hong Kong SAR 999077 China; ^3^ Shenzhen Research Institute City University of Hong Kong Shenzhen Guangdong 523808 China; ^4^ State Key Laboratory of Bioreactor Engineering Shanghai Key Laboratory of New Drug Design School of Pharmacy East China University of Science and Technology Shanghai 200237 China

**Keywords:** drug combination, high‐throughput screening, microdroplet array, microfluidics, zebrafish

## Abstract

Large‐scale screening of molecules heavily relies on phenotyping of small living organisms during preclinical development. However, deep profiling candidate therapeutics on whole animals typically requires laborious manipulations and anesthetic treatment using traditional techniques or automated tools. Here, a novel fish capsule system that combines automated zebrafish encapsulating technology and droplet microarray strategy for in vivo functional screening of mono/polytherapies is described. This platform enables automated, rapid zebrafish orientation and immobilization in agarose to generate large‐scale fish capsules by using a microfluidic device. Based on the effect of discontinuous dewetting, the prompt trapping of fish capsules in the aqueous arrays is successfully demonstrate. This system provides the capability to integrate pharmaceutical treatments with real‐time multispectral microscopic imaging in a simple, pipetting‐free and highly parallel manner. Coupling with machine learning algorithms, a small library of compounds is screened and analyzed, and clues about how to exploit compound combinations as therapeutic candidates are obtained. It is believed that this proposed strategy can be readily applied to multiple fields and is especially useful in the exploration of combinatorial drugs with limited amounts of samples and resources to accelerate the identification of novel therapeutics for precision medicines.

## Graphical abstract

A novel fish capsule system, based on automated zebrafish encapsulating technology and microdroplet array strategy, is developed to enable large‐scale immobilization of oriented zebrafish larvae and compound parallel addition. Coupling with bioinformatic analysis and machine learning algorithms, this system is successfully demonstrated for in vivo high‐throughput functional screening of mono/polytherapies.

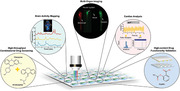

## 1 Introduction

Polytherapy, utilizing multiple drugs to achieve higher therapeutic efficacy and fewer side effects is widely used in the clinical treatments of many diseases, including central nervous system (CNS) disorders.^[^ ^1^, ^2^, ^3^, ^4^ ^]^ Even though the traditional guidelines of the treatments recommend monotherapy, there is a growing consensus that most of the patients finally receive polytherapy.^[^ ^5^, ^6^, ^7^ ^]^ Apparently, discovering optimal drug combinations has been of interest for a long time.^[^ ^8^ ^]^ Given that treatments of complex diseases such as CNS disorders will probably require system‐modulating therapeutics, it is difficult to identify with traditional in vitro readouts.^[^ ^4^ ^]^ Thus, in vivo functional platforms are extremely important for high‐throughput screening of large libraries of combinatorial compounds.

Unfortunately, despite recent efforts and success that have been made in the development of technologies for large‐scale drug combination screening based on chemical binding tests, cell culture assays and tissue/organoids evaluations, in vivo combinatorial drug screening is rarely reported.^[^ ^9^, ^10^, ^11^ ^]^ This is due in large part to the time, space, technical and financial demands of high‐throughput in vivo screens with traditional rodent animal models. Zebrafish, a widely accepted vertebrate model for biomedical investigations, is particularly useful for in vivo chemical evaluations and drugs screening by virtue of its small size, high reproduction rate, ease in genetic manipulation and optical transparency at the larval stage. High‐throughput morphological or behavioral assays using zebrafish have also been successfully demonstrated in large‐scale neuroactive compound discovery.^[^ ^12^, ^13^, ^14^, ^15^ ^]^ However, there are still some key challenges to be addressed before one can fully realize the potential of systematic profiling in zebrafish for combinatorial drug screening. Although significant efforts have been devoted to developing functional techniques to manipulate zebrafish for specific organ observation, high cost, complex external systems and limited throughput have highly restricted their applicability. For example, a powerful platform (vertebrate automated screening technology, VAST) was developed to enable automatic immobilization and orientation of zebrafish based on a rotating glass capillary and a complicated transportation and control system.^[^ ^16^ ^]^ However, this platform is highly costly, technologically challenging and is only capable of manipulating a limited number of animals. Recently, a contactless technique has been proposed to enable morphological phenotyping of zebrafish based on acoustofluidic rotational tweezing.^[^ ^17^ ^]^ Even though it presents a functional platform for rapid manipulation and evaluation of zebrafish larvae, this system requires anesthetic treatment to the animals, which has been concerned to induce aversion, neurotoxic damage in the immature brain, reduction of heart rate, and consequently high mortality, especially during long‐term exposures, and may impose artifacts in drug discovery.^[^ ^18^, ^19^, ^20^ ^]^ Reported alternative platforms for behaving whole organism screening are based on hydrodynamic force using microfluidic devices, such as the Zebrafish Entrapment By Restriction Array (ZEBRA) device and the “Fish‐Trap” system.^[^ ^21^ ^]^ Such systems are suitable for animal manipulation and drug perfusion; however, they are not compatible with high‐throughput screening the combinations of drugs in large compound libraries owing to the interconnected arrays inside the microfluidic chips. In this regard, there is an unmet need to develop a cost‐effective, simple and highly functional system for automated immobilization, orientation and evaluation of zebrafish in rapid identifying the system‐modulating combinatorial therapeutics.

Herein, we demonstrate a fish capsule (FC) system that combines automated zebrafish capsulation technology and droplet microarray strategy to enable in vivo high‐throughput screening of polytherapy. Using the microfluidic device, similar to the system that we reported recently,^[^ ^20^, ^22^ ^]^ rapid zebrafish orientation and immobilization in agarose is conducted simultaneously. This technique is straightforward, stable and time‐saving in the process compared to the traditional agarose confinement.^[^ ^23^ ^]^ Then, a simple collection of the fish capsules and capsules trapping in the aqueous arrays are demonstrated. The formation of aqueous arrays was achieved by the design of the superhydrophilic spots separated by superhydrophobic barriers and the effect of discontinuous dewetting. Specifically, the fish capsule containing a whole animal could be seen as a large particle, which is easy to be transferred, evaluated and quantified without any anesthetic treatment in our system. Along with multispectral microscopic imaging, this FC system could be applied at various imaging resolutions for real‐time visualization of both the whole body and any specific organ. To validate the functionality of our FC system, we demonstrate a combinatorial screening of a set of neuroactive compounds been combined by another set of cardiovascular drugs by systematic profiling in zebrafish. In this proof‐of‐concept screen, the parallel additions of compound libraries were performed via the sandwiching method. As a result, we found four pairs including clozapine combined with disulfiram/amitriptyline and sertraline combined with phenytoin/amitriptyline showed a promising effect in neural modulation in the brain without cardiac side effects. In addition, we investigated the synergism of the pairs from the dose–response matrix. Notably, to our best knowledge, it is the first time to dissect the functionality of these combinatorial drugs in such in vivo screens.

We believe that our FC system can narrow the gap between in vivo large‐scale screening and individual laboratories with limited resources, thereby expanding the application of the zebrafish model in various biomedical and biological fields, and in particular for accelerating combinatorial drug discovery and further simplifying screens of personalized therapies in animal models.

## 2 Results

### 2.1 Design of the “Fish Capsule” System

**Figure 1:**
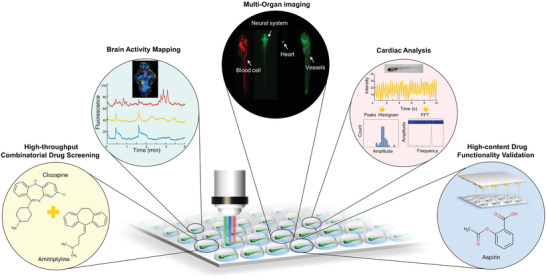
High‐throughput, high‐content, multifunctional screens enabled by the fish capsule (FC) System. The FC system enables rapid capsuling a large scale of zebrafish larvae with ordered orientation for further on‐chip brain‐wide activities mapping, cardiac function analysis, multiorgan imaging and parallel compound addition for high‐content drug functionality validation and high‐throughput combinatorial drug screening using microdroplet arrays.

**Figure 2:**
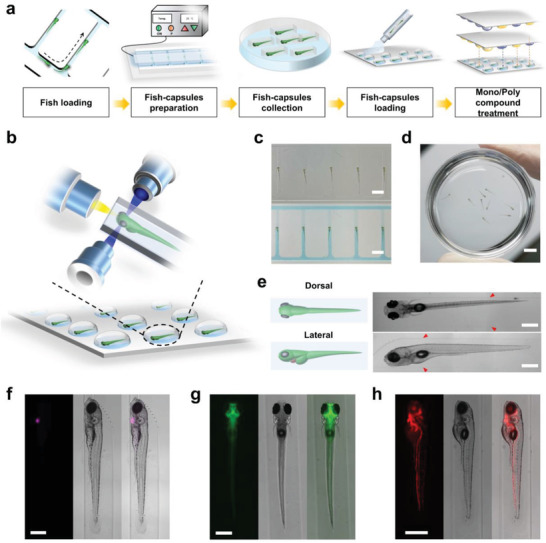
Rapid generation of zebrafish capsules for specific organ microimaging. a) Schematic diagram showing the process of the fish capsule (FC) system. First, zebrafish larvae would be loaded, trapped and oriented based on a hydrodynamic force via utilizing a microfluidic chip. After immobilization of the oriented larvae, the chip would be fulfilled agarose and be cooled down to a specific temperature to generate large‐scale zebrafish capsules. Then the fish capsules would be easily collected and loaded on a superhydrophilic/superhydrophobic array. Last, a sandwiching method was applied to enable parallel mono/poly compound addition for high‐throughput screening. b) Schematic describing the large‐scale imaging of zebrafish larvae with microscopy. c) Immobilization of multiple awake larvae in a microfluidic chip with a dorsal‐up orientation in water (top) or agarose (bottom). Scale bar, 2 mm. d) Multiple zebrafish capsules were collected in a container. Scale bar, 4 mm. e) Schematics (left) and images (right) showed a larva with dorsal (top) and lateral (bottom) orientation, respectively. The red arrows indicated the interfaces of the capsules. Scale bar, 0.5 mm. Representative fluorescent/white‐field/merged images of heart, brain and blood cells in the Cmlc2:eGFP f), Elavl3:H2B‐GCaMP6f g), and Gata1:dsRed h) transgenic animals, respectively. Scale bar, 500 µm.

To enable high‐throughput, high‐content, multifunctional screens using zebrafish, we developed a fish capsule (FC) system, evolving from the existing microdroplet array technology to expand its capabilities, that can be used to immobilize oriented animals for a broad range of multiorgan imaging, specific organ function analysis, and large‐scale screening applications (**Figure** **1**). First, we combined microfluidic techniques and a hydrogel encapsulation method to rapidly generate fish capsules (**Figure** **2**a; Movie S1, Supporting Information). Unlike the conventional zebrafish larvae immobilization strategies using a rigid gel or a glass capillary,^[^ ^16^, ^23^, ^24^ ^]^ which were neither tedious nor difficult in animal confinement for optimal microscopic imaging at the desired viewing angle, the FC system utilized a microfluidic chip to allow automatic and anesthetic‐free loading, trapping, orientating and immobilizing tens of hundreds of fish larvae via hydrodynamic force controlling (Figure 2b–d). The microfluidic chip was fabricated via high‐precision computer‐numerical‐control (CNC) machining and polydimethylsiloxane (PDMS) demolding (Figure S1, Supporting Information). Generally, owing to the flow resistance distribution in the vertical trapping channels with baffle design in each outlet, the bulk stream in the horizontal flow channel would guide the larvae to be trapped in the trapping channels one by one (Figures S2 and S3 and Movie S2, Supporting Information). The larvae loading and transportation was enabled via a homemade handling system that employed digitally controlled syringe pumps, microfluidic valves and thermal imaging sensors (Figure S4, Supporting Information). After positioning the larvae in the chip, two major orientations of the trapped animal, lateral or dorsal, could easily be achieved by simple adjustments of the flow rate and thus rendering the capability to microimaging internal systems including the central nervous system and cardiovascular system (Figure 2e). Coupling with the well‐developed and abundant resources of zebrafish transgenic lines (including elavl3:H2B‐GCaMP6f, Gata1:dsRed and Cmlc2:eGFP), our system was readily to be utilized in multiple organ investigations (Figure 2f–h). To further facilitate the long‐term immobilization of the larvae without continuous hydrodynamic force, we then integrated a temperature controllable hotplate with the microfluidic system to finally capsule the oriented animals within the agarose‐based hydrogel in the microchannels of the chip (see the Experimental Section for details). A similar strategy has been recently demonstrated on different small animal models.^[^ ^25^, ^26^ ^]^ The hydrogel remains in the liquid phase at room temperature (28 °C) and solidifies when the surrounding temperature is briefly below 25 °C. Subsequently, many agarose capsules containing oriented animals could be simply cut down and collected like large particles when the PDMS layer of the microfluidic device was gently removed (Figure 2d). The microfluidic manipulation and the short‐term temperature fluctuation in hydrogel did not affect the health of the larvae, which was consistent with our further assessment. Using the fish capsules, our system could be utilized in specific organ investigations.

**Figure 3:**
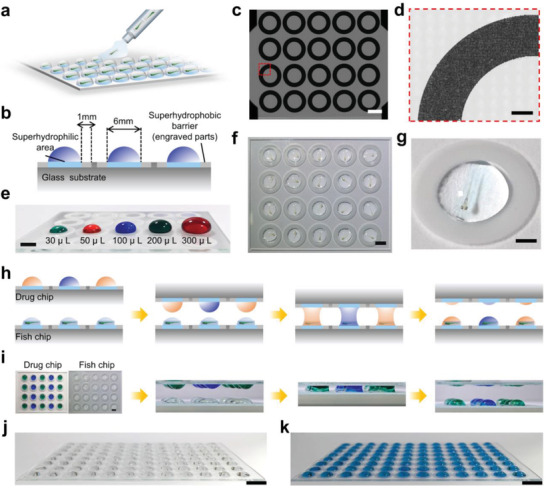
Parallel compound addition using microdroplet arrays. a) Schematic representation of spreading zebrafish capsules using the effect of discontinuous dewetting. b) Schematic describing the structure of the droplet array chip. c,d) Representative microscopic images of microstructure of droplet array chip. Image in (d) was zoomed in from the dotted box in (c). Scale bar, 5 mm c) and 0.5 mm d). e) An image exhibited extremely high hydrophobic properties with a static water contact angle (WCA) up to 110° when the capacity to carry liquid volumes up to 300 µL. Scale bar, 4 mm. f) A representative image showed an array of droplets each containing a single fish capsule. Scale bar, 5 mm. g) An enlarged photograph showed an immobilized fish capsule with dorsal‐up orientation in a droplet. Scale bar, 2 mm. Schematics h) and images i) of parallel compound addition via sandwiching method. Images of fish capsules array in a 96‐well format before j) or after k) parallel compound addition. Scale bar, 10 mm.

In the FC system, the fish capsules with oriented animals were then separated onto a droplet array to enable high‐throughput screening (**Figure** **3**a; Movie S3, Supporting Information). In this proof‐of‐concept study, a glass slide consisting of arraying circles, each containing a superhydrophilic spot with 6 mm diameter and 1 mm wide superhydrophobic border, was fabricated (Figure 3b). To prepare the droplet arrays, patterns of circles with defined sizes were engraved by laser in a mass‐produced way (Figure 3c,d). The patterned circles were further coated with a commercial superhydrophobic solution by parallel dipping after plasma treatment (the spot in the middle of each circle was superhydrophilic after plasma treatment). Due to the laser engraved circles, these two opposite solutions were well restricted in each spot and border. The treated circles exhibited extremely high hydrophobic properties with a static water contact angle (WCA) up to 110° (Figure 3e). After air‐drying, arrays of separated droplets could easily be generated when aqueous solutions was applied onto modified slides as a result of discontinuous dewetting, which have been demonstrated in the rapid spreading of solutions, cells and eggs.^[^ ^27^, ^28^, ^29^ ^]^ Based on the effect of discontinuous dewetting, an array of droplets, each containing a single fish capsule that we collected above was generated by using a pipette manually or using a computer‐controlled syringe pump and motorized *X*–*Y* stage robotically (Figure S5, Supporting Information). By moving the pipette with a constant speed, each capsule would be pushed out and suspended on the tip of the pipette by gravity. Then the capsules would be deposited onto the superhydrophilic spot one after another. In this way, each fish capsule would be trapped and confined effectively to the droplet on the superhydrophilic spot for further specific organ microscopic imaging (Figure 3f). The height of the microdroplet played a critical role in strictly immobilizing zebrafish capsules in the droplet. To avoid drying and optical distortion during long‐term evaluation, the droplet volume was on average of 10 µL, which well fitted the spot dimensions and was suitable to confine each capsule (Figure 3g). This liquid volume not only made the height of each microdroplet slightly lower than the height of the capsule to enable strict immobilization but also was relatively low when compared to the classic volume that was used in the traditional screening of zebrafish larvae, ranging from hundreds of microliters to tens of milliliters in multiwell plates or recently developed microfluidic systems. It is especially essential for screens of the highly expensive compounds, e.g., neuroactive compounds or anticancer drugs. Moreover, this strategy enabled multiplexing assays of different drugs in a sufficient number of animal replicates in a device, which was always problematic in traditional microfluidic systems (Figure 3h,i). To further improve the throughput of our FC system in practical screening applications, a 12 × 8 array system was also fabricated to be compatible with the industrial standard 96‐well plate format (Figure 3j,k).

We next assessed the health of 36 larvae based on functional and morphological criteria after running through our FC system. In a 48 h health assessment, no significant difference in the heartbeats, blood flow rates or morphological abnormalities was detected between the screened larvae and freely moving animals (Figure S6, Supporting Information). Morphological abnormality of the larvae across the examined population at first 12 h was 0% and 2.8 ± 2.5% for FC system and manual embedment, respectively. And the survival rate of the animals released from FC system was 91.7 ± 3.4% at 48 h evaluation, which was much higher than that in the manual processing group (61.1 ± 5.1%). It suggested that the FC system not only induced a considerably faster manipulation of larvae but also largely reduced the damage rates in agarose capsuling compared with the manual process (Figure S6d, Supporting Information). Importantly, the larvae that were carefully released from the system showed good locomotion behaviors after running through a complete experimental cycle in the FC system (Figure S7, Supporting Information). To further validate the biocompatibility of our technique while superhydrophobic coatings (slightly toxic) were involved in our system, we monitored the physiological changes of the zebrafish larvae. As shown in Figure S8 (Supporting Information), compared to the animals that freely moved in a culture dish, no detectable damage was found in the larvae that were trapped in the microdroplets during a 48 h toxicity test. Collectively, these results show that our system has enabled the encapsulation of living zebrafish larvae with desired orientation rapidly and safely, which could further benefit the in vivo large‐scale screening.

### 2.2 Parallel Compound Addition and Multiple In Vivo Phenotyping

To validate the functionality of our FC system, we first evaluated the effects of monotherapy within a small group of neuroactive compounds and another set of cardiovascular drugs by systematic profiling in zebrafish, respectively (Tables S1 and S2, Supporting Information). In this study, a sandwiching method was used to enable parallel additions of compound libraries by sandwiching the droplet arrays containing zebrafish capsules with another slide containing the compounds as shown in Figure 3h. A motorized 3D stage was equipped to perform alignment and movement between the inverted drug slide and the downside FC to allow parallel additions of the compounds (Figure 3i). Then the sandwiched arrays were kept in an incubator for 1 min to ensure the diffusion of the compounds into each droplet (Figure S9 and Movie S4, Supporting Information). Predominantly, owing to the frosted superhydrophobic barriers, there is no cross‐contamination during drugs delivery (Figure S10, Supporting Information). Moreover, this sandwich strategy for large‐scale compound addition did not induce any stress to the fish capsules and thus did not induce any artifacts to the final compound evaluation (Figure S11, Supporting Information).

**Figure 4:**
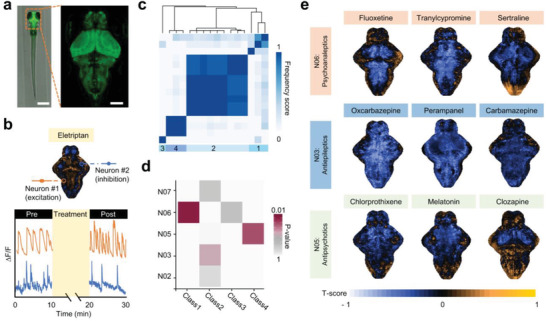
Functional classification of CNS drugs based on brain activities mapping using the fish capsule (FC) system. a) Representative brain image of a larva (from Elavl3:H2B‐GCaMP6f transgenic line) trapped in the system. Scale bar, 0.5 mm (left), 100 µm (right). b) Representative fluorescence traces of selected neurons as indicated by circles the *T*‐score brain activity map (T‐score BAM) of eletriptan, an antimigraine, showing the spiking activity of each neuron. c) Consensus clustering identified 4 phenotypic BAM classes. The heat map was the consensus matrix illustrating the frequency of the scenario that two compounds in a pair were clustered together. The color of the heat map is proportional to the frequency scores of the consensus matrix, ranging from 0 to 1. d) A heat map illustrating statistical associations between the Anatomical Therapeutic Chemical (ATC) categories and identified T‐score BAM‐classes. Color coding is based on *p*‐values derived from the hypergeometric tests (red: lower *p*‐values or more significant association; gray: higher *p*‐values or less significant association). e) The *T*‐score BAMs of the drugs in the signature subgroup of class 1 (significantly associated with N06: Phsychoanaleptics ATC category), class 2 (significantly associated with N03:Anti‐epileptics ATC category), and class 4 (significantly associated with N05:Antipsychotics ATC category), respectively.

We then utilized our FC system to evaluate the effects of a small group of neuroactive compounds on the brain activities in zebrafish larvae using the transgenic line elavl3:H2B‐GCaMP6f, which is genetically encoded a calcium‐sensitive fluorescent indicator (**Figure** **4**a).^[^ ^30^ ^]^ Coupling with high‐speed microscopic imaging, the brain‐wide activities of these larvae could be recorded in single‐cell resolution (Movie S5, Supporting Information). To quantitatively analyze the drug‐induced changes of the brain activities, we generated a *T*‐score brain activity map (*T*‐score BAM) for each animal in response to the corresponding drug treatment as we reported recently.^[^ ^22^ ^]^ The zebrafish larva was encapsulated in our FC system with dorsal‐up orientation, which enabled calcium imaging in multiple focal planes along the *Z*‐axis (ventral direction) before and after drug additions. In this study, five brain activity maps (BAMs) were acquired from five droplets by counting the calcium transients and then used to generate the significance scores via statistical analysis with a *T*‐score test at every single unit (Figure 4b). A unique *T*‐score BAM for each drug was constructed by projecting the significance score of each unit (≈15 µm) to the final map. In this work, 16 clinically used CNS drugs including a variety of known therapeutic uses shown in the World Health Organization (WHO) Anatomical Therapeutic Chemical (ATC) classification system, were tested (Table S1, Supporting Information). Visually, most of the drugs induced acute changes in the brain activities as shown in their *T*‐score BAMs. For example, clozapine, an antipsychotic drug,^[^ ^31^ ^]^ robustly increased the neural activities in the forebrain, cerebellum and hindbrain; sertraline and fluoxetine, selective serotonin reuptake inhibitors,^[^ ^32^ ^]^ both used as antidepressant drugs, resulted in increased calcium fluctuations in the forebrain and partials of the hindbrain but slightly silenced the activities elsewhere in the brain (Figure 4e). To further extract the intrinsic coherence among all the *T*‐score BAMs, we first applied principal component analysis (PCA) to reduce the dimensionality and noise (Figure S12, Supporting Information). Then a consensus clustering method based on hierarchical clustering was employed to dissect the phenotypic diversity based on the principal components (PCs) (Figure 4c). Four optimal phenotypic *T*‐score BAM classes were identified in this study. Among them, three *T*‐score BAM classes namely class 1, class 2 and class 4, were further found to be statistically associated with the drugs’ therapeutic categories including the antidepressant drugs (N06), antiepileptic drugs (N03) and antipsychotic drugs (N05) as determined by the WHO ATC classification system via hypergeometric tests (Figure 4d,e). Collectively, these results indicated that our FC system could be applied in the monotherapy screening of neuroactive drugs.

**Figure 5:**
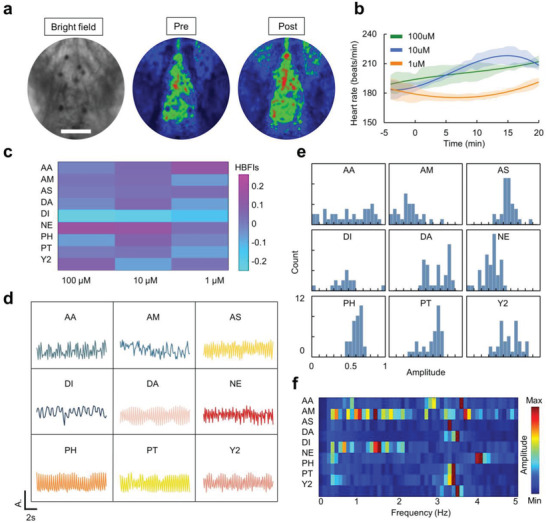
High‐throughput screening of cardiac drugs based on label‐free fingerprints. a) A representative image of heart beating recorded from a wild‐type larva and its corresponding heat maps before and after norepinephrine treatment. Scale bar, 100 µm. b) Heart rate fluctuation with norepinephrine treatment at doses ranging from 1 × 10^−6^ to 100 × 10^−6^ m. Error bars indicate standard error of the mean (s.e.m.), *n* = 5. c) A heat map showed the HBFIs (the heartbeat fluctuation index) of the cardiac drugs with different concentrations. d) Representative cardiac signals recorded using the fish capsule (FC) system in responses to the drugs additions. e) Histogram of the representative cardiac signal peaks under different conditions. f) Spectrum analysis of the representative cardiac signals using fast Fourier transform (FFT).

Next, we employed our FC system in evaluating the effects of another group of compounds on cardiac physiology in larval zebrafish (Movie S6, Supporting Information). As a proof of concept, nine drugs reported to induce physiological influences on cardiac cells in vitro or in vivo were tested in this study (Table S2, Supporting Information). Real‐time recordings of heart beating were performed in tens of larvae before and after the drug additions using the wild‐type animal or the cmlc2 transgenic line (Figure S13, Supporting Information). The majority of the drugs did, indeed, induce acute changes in zebrafish cardiac function, e.g., norepinephrine, a neurotransmitter which had been shown to increase cardiac output through direct *β*1‐agonist inotropic effect and*α*‐agonist‐mediated restoration of diastolic arterial pressure,^[^ ^33^ ^]^ significantly increased the heart rates with the doses of 10 × 10^−6^ and 100 × 10^−6^ m (**Figure** **5**a,b; Figure S14, Supporting Information). For quantitative analysis of the long‐term heart beating in the animals, we used an index, the heartbeat fluctuation index (HBFI), to further extract the influences of the drugs on heart rates. Based on the HBFIs, we generated a heat map to visually reflect the regulation of the heart rate from the drugs (Figure 5c), e.g., disulfiram, an alcohol withdrawal drug which had been reported to significantly reduce systolic and diastolic blood pressure in rats,^[^ ^34^ ^]^ exhibited an inhibitory effect on heart rate with all the concentrations (−0.24 HBFI, −0.21 HBFI, and −0.14 HBFI in 100 × 10^−6^, 10 × 10^−6^, and 1 × 10^−6^ m doses, respectively); norepinephrine increased the heart rate in high doses (0.16 HBFI, 0.14 HBFI in 100 × 10^−6^ and10 × 10^−6^ m concentrations, respectively). To deeply dissect the cardiac activities in response to pharmaceutical treatments, we analyzed the raw signals from the aspects of frequencies, phases, and amplitudes (Figure S15 and Table S3, Supporting Information). The representative signal envelopes were also extracted from the original recordings to show in vivo dynamic responses of the heart to the drugs (Figure 5d). For example, disulfiram caused a dramatic reduction in the signal amplitude; Y‐27632, an inhibitor of Rho‐associated protein kinase,^[^ ^35^ ^]^ induced large peaks which were spaced by multiple small peaks. The peak amplitude histogram (Figure 5e) and spectrum pattern from the fast Fourier transform (FFT) (Figure 5f) also showed some characteristic features of the cardiac signals that were specific to certain drugs. Basically, using the HBFIs (features in the temporal domain), the envelops (features in the amplitude domain) and the FFT patterns (features in the frequency domain), we successfully provided an in vivo fingerprint for each drug's therapeutic effects in cardiac function (Figure 5).

### 2.3 Combinatorial Drug Screening Using the FC System

**Figure 6:**
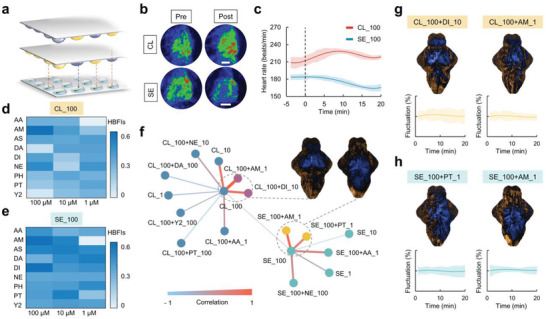
Large‐scale drug combination assay using the fish capsule (FC) system. a) Schematic illustration of the combinatorial drugs screening process. b) The representative heat maps showed the cardiac effects of clozapine (CL) and sertraline (SE), respectively. Scale bar, 50 µm. c) The statistic results of the heart rate recording with CL or SE treatment at doses of 100 × 10^−6^ m. d,e) Heat map of each pair of the combinatorial drugs’ heart beat fluctuation indexs (HBFIs). f) Visualization of the correlation between the brain activities maps of the combinatorial drugs and the monotherapy based on their Pearson correlation coefficient. Two clusters of the BAMs were indicated by the dotted circles, which represented a high correlation (>0.7). The BAM patterns derived by taking the mean of all clustered drugs in each circle. g,h) Identified drug pairs (including 100 × 10^−6^ m CL combined with 10 × 10^−6^ m disulfiram (DI); 100 × 10^−6^ m CL combined with 1 × 10^−6^ m amitriptyline (AM); 100 × 10^−6^ m SE combined with 1 × 10^−6^ m phenytoin (PT); 100 × 10^−6^ m SE combined with 1 × 10^−6^ m AM reduced the cardiac side effects of CL g) and SE h), respectively, but induced similar *T*‐score BAMs as their monotreatment. Error bars indicate standard error of the mean (s.e.m.), *n* = 5.

Parallel additions of compounds using the sandwiching method showed great advantages in drug combinational screening and had been frequently demonstrated in in vitro evaluations.^[^ ^11^ ^]^ Nevertheless, owing to the laborious manipulation of the animals, its utility in in vivo combinatorial drugs screening, especially targeted specific organ functions, has been rarely demonstrated. As we demonstrated above, our FC system enabled large‐scale oriented‐zebrafish capsules in recording a featured response with sufficient characteristics to capture the distinct pharmaceutical efficacy of different drugs in multiple organ functions. Hence, coupling with our FC system, the sandwiching strategy was next applied in implementing a cross‐systematic combinatorial drugs screening using zebrafish (**Figure** **6**a).

Considering neurological drugs are often associated with various side effects, e.g., clozapine, an antiepileptic drug which commonly induces side effects such as tachycardia, myocarditis and cardiomyopathy, we used the FC system to access the cardiac effects of a small group of neuroactive compounds. As shown in Figure 6b,c, within 20 min after drug treatments, 100 × 10^−6^ m clozapine induced a 13.1 ± 4.2% increase in the zebrafish heart rate; conversely, 100 × 10^−6^ m sertraline reduced the animals’ heart rate by 15.9 ± 1.9% (Figure S16, Supporting Information). Prominently, both of these observations were consistent with the reports of previous studies.^[^ ^36^, ^37^, ^38^ ^]^ To further demonstrate the functionality of the system in investigating the interaction of combinatorial drugs, we parallelly loaded the combinations of neuroactive compounds and cardiac drugs to each microdroplet and analyzed their regulations of heart activities in zebrafish. Visually, several drug combinations showed powerful alterations of the cardiac side effects of the clozapine and sertraline (Figure S17, Supporting Information). To quantitatively compare the heart rate fluctuations, we then applied the HBFI analysis on the recorded results to each drug pair. In this test, 100 × 10^−6^ m clozapine respectively paired with 1 × 10^−6^ m amitriptyline/1 × 10^−6^ m aminobutyric‐acid/10 × 10^−6^ m disulfiram/100 × 10^−6^ m dopamine/100 × 10^−6^ m Y‐27632 had shown low HBFIs (lower than 0.03) among the total 54 combinations (Figure 6d). And 100 × 10^−6^ m sertraline respectively paired with 1 × 10^−6^ m amitriptyline and 1 × 10^−6^ m phenytoin were also revealed to be the promising combinations that induced relatively low HBFIs (lower than 0.03) (Figure 6e). To quantitatively compare the effectiveness of these drug pairs to the monodrug, we performed a similarity analysis based on their Pearson correlation coefficient between each *T*‐score BAMs (Figure 6f). Interestingly, several drug pairs among the screened combinations showed high correlation coefficients in *T*‐score BAMs, indicating such pairs might have homogeneous effects on neural modulation in the brains of the animals. In particular, compared to the monotherapy, certain drug pairs induced similar *T*‐score BAMs (Figure 6f–h). For example, 100 × 10^−6^ m clozapine followed with 10 × 10^−6^ m disulfiram, also resulted in decreased neural activity in parts of the midbrain but marked increase in calcium transients elsewhere in the brain (Figure 6g); 100 × 10^−6^ m sertraline paired with 1 × 10^−6^ m amitriptyline, showed similar upregulation in neural activities in the forebrain and partials of the hindbrain (Figure 6h). Collectively, as visualized in the correlation network diagram and the *T*‐score BAMs, the typical pairs showed high similarity, which further confirmed that these drug combinations have similar influences on the brain functions.

## 3 Discussion

We have previously validated the functionality of the microfluidic system for automatic larvae orientation, specific organ imaging and acute compound treatment.^[^ ^20^, ^22^ ^]^ However, the continuous flow in such systems makes it difficult for sample transfer, large‐scale combinatorial drugs screens and system setup. Moreover, the liquid volume used in the previous platforms was quite large, particularly in the long‐term and large‐scale screens. In this study, we have demonstrated a fish capsules (FC) system that integrates microfluidic manipulation, hydrogel‐based encapsulation and superhydrophilic‐superhydrophobic micropatterning for high‐throughput in vivo phenotyping, especially in combinatorial drugs screening. The FC system makes it simple for sample transfer, platform setup and low‐volume compound consumptions in long‐term screens. Specifically, using the hydrodynamic force that resulted from the unique design of our microfluidic device, the FC system enabled the automatic orientation and immobilization of zebrafish larvae when coupling with controllable hydrogel encapsulation. Furthermore, the self‐formed microdroplet array based on discontinuous dewetting effect was employed in the one‐step spreading of fish larvae and parallel addition of compounds, which could replace the highly repeated pipetting operations in large‐scale assays. In our platform, the incubation volume of a single larva in each microdroplet was as low as 10 µL. To our best knowledge, this is the first reported technique that enabled screening of oriented zebrafish larvae in such relatively low liquid volume. Collectively, this significant decrease in the sample consumption and workload would make laboratories or R&D groups in the companies with limited screening resources possible to perform large‐scale in vivo assays in an affordable and time‐efficient way.

In the majority of plate‐based zebrafish screening, several larvae would be located in one well for reducing the compound consumption to a milliliter level.^[^ ^12^, ^13^ ^]^ However, the random distribution of the larvae in the well has a high possibility to induce different orientations and sporadic movements, which makes it challenging for specific organ microimaging.^[^ ^16^ ^]^ This study showed that automatic encapsulation of zebrafish with the dorsal or lateral position was successfully demonstrated using the FC system. Although dorsal and lateral orientations have rendered the capability to image almost all the major organ systems including the brain, heart, vascular and blood cells (Figure S18 and Movies S5–S7, Supporting Information), other specific viewing angles were ready to be achieved by using our FC system, e.g., for visualization of the fluorescent markers or other internal targets and laser ablation of cells and tissues in animals.^[^ ^26^ ^]^ It largely simplifies the specimen loading, trapping and orientating for gel‐based in vivo high‐resolution microscopic recording and evaluation of specific organs compared to previous manual strategies.^[^ ^23^, ^24^, ^39^, ^40^ ^]^ Importantly, this technique has enabled the encapsulation of oriented animals in an anesthetic‐free way. This feature would be challenging for previous gel‐based animal immobilization due to the spontaneous behavioral reactions during manipulations.^[^ ^3^ ^]^ Particularly, without anesthetic treatment, our platform is highly suitable for the evaluation of the neural systems and cardiovascular systems in animals, which is also difficult for other proposed microfluidic platforms.^[^ ^16^, ^17^ ^]^ Moreover, by virtue of the large‐scale oriented animals in the FC system, our technique was ready to be easily adopted in screens of highly hydrophobic compounds, large molecules and proteins by microinjecting into the targeted organs, e.g., yolk sac, brain, heart or the circulation (Figure S19, Supporting Information).

Using our FC system, we go on to demonstrate how the oriented whole‐organism array can be utilized to enable multiorgan evaluations in combinatorial drugs screening. The common practice to encapsulate an oriented larva in a rigid gel requires laborious manual operations, which results in unreliable readouts and further limits its applications in the screen of the mono/polytherapies. The FC system enables automatic orientation and gel‐encapsulation of animals in large‐scale, making it suitable for in vivo high‐throughput screening. In this demonstration, a functional fingerprint‐based strategy was also employed to enable label‐free pharmacological identifications including cardiac drugs and neuroactive drugs. Notably, several drug pairs were also found to induce robust neural regulations as similar as the monotherapy but without obvious cardiac side effects. For example, clozapine paired with amitriptyline or disulfiram showed stabilizing heart rates when compared with only clozapine treatment; and both induced obvious inhibition in parts of the midbrain but marked excitation elsewhere in the whole brain. Although there are some clinical case reports using the drugs combinations mentioned above to treat psychotic diseases and the clinical discussion focusing on the potential influences of the selective serotonin reuptake inhibitors on the pharmacokinetics of the tricyclic antidepressants has also been reported,^[^ ^41^, ^42^ ^]^ such in vivo investigations of systematic pharmacology of the certain combinatorial drugs had not been demonstrated prior to our study. By classifying single/combinatorial compounds on their actual in vivo alterations in their neural and cardio readouts using bioinformatic approaches, we could identify highly effective candidates for polytherapy without any prior knowledge of their chemical structures and molecular targets. In addition, our FC system and the screening strategy presented here are ready to be further refined in several ways. For example, deep learning algorithms could be applied in the analysis of the multidimensional data including the time‐varying activities of the 3D whole organs and single cells in specific systems;^[^ ^43^ ^]^ more advanced whole‐organ imaging and registration techniques could also be engaged in our system to assess the compounds capable in affecting specific cell populations.^[^ ^44^, ^45^ ^]^ We believe that our FC system shows great potential as the basis for cost‐ and time‐efficient animal manipulations, transportations and evaluations in zebrafish studies, and creates a new paradigm for combinatorial drug screening, personalized therapy evaluation and systematic physiology investigations.

## 4 Experimental Section

### Zebrafish Line

The wild‐type zebrafish and the transgenic zebrafish line elavl3:H2B‐GCaMP6f, Kdrl1:eGFP, Gata1:dsRed and Cmlc2:eGFP were maintained in aquaria under standard laboratory conditions (at 28 °C under a cycle of 14 h light, 10 h dark). Larvae of 6–8 dpf were used in the combinatorial drug screening experiments. All animal work was carried out with prior approval from the animal ethical committee of Sun Yat‐sen University and was in accordance with local animal care guidelines.

### Microfluidic Chip

The microfluidic chip was one of the key components of the fish capsule (FC) system. It was used to enable the orientation and immobilization of zebrafish for further preparation of zebrafish capsules. The microfluidic chip with specific channels and structures was designed to trap larvae on a large scale by combing hydrodynamic force. The array of chambers for trapping zebrafish larvae were evenly distributed between two parallel main channels (900 µm wide and 800 µm high) (Figure S1, Supporting Information). The zebrafish were loaded with specific direction (heads facing forward) into the trapping chamber via an automated loading system as previously described (Figure S2, Supporting Information). The height of the core structure of the trapping chamber decreased in a specific arc from 800 to 250 µm, which allowed a considerable adaptation to the physiological structure of a fish head. This structure also protected the animals from injury. After orientation and immobilization of the larvae, agarose gel would be induced into the chip by simply switching the inlet from water to gel. Importantly, the moderate design of each main trapping chamber not only accommodated the entire larval body but also reserved sufficient space for the flow of agarose so that the zebrafish can be effectively encapsulated in the gel (Figure 2). To accommodate feature sizes of both micrometer and millimeter scales in the design, a high‐precision computer numerical control (CNC, Dingsheng CNC Equipment Co., Ltd.) machining method was utilized to fabricate the mold of the microfluidic chip (Figure S3, Supporting Information). First, a positive mold of the microfluidic design was machined on a plain copper substrate with 30 µm resolution. After soaking in 100% alcohol overnight, the mold was rinsed with deionized water a couple of times and dried in the air. Then polydimethylsiloxane (PDMS) was used to make the chips with flow channels by replicating molding from the copper molds. PDMS was poured onto the mold and baked in an 80° oven for 4–6 h after completely removing the air bubbles in a vacuum chamber. Then the cured PDMS layer was demolded for further punching to form the inlets and outlets. Last, the modified PDMS layer was temporarily bonded to the glass substrate to make the final microfluidic chip that used in the FC system.

### Microfluidic Handling of Larval Zebrafish

The automatic loading and transportation of zebrafish larvae were enabled via a homemade external system based on an open‐source electronics platform coupling with several microflow valves, syringe pumps and thermal imaging sensors (Figure S4, Supporting Information). Briefly, an Arduino board (Arduino UNO R3, a microcontroller board based on the ATmega328P) was applied here to digitally control the pumps and the electromagnetic fluidic valves (WK04‐010‐0.5/1‐NC; Wokun Technology). Two syringe pumps (v2, Guanjie Technology) were used to load the zebrafish larvae from a reservoir into the fluidic circuitry. A sensing box based on thermal imaging sensors (WX200, Weixin Vision Technology Co., Ltd.) was developed to detect the fish loading and the direction of the animal head, which was then used as the trigger signal to actuate the corresponding pumps and valves. This automatic direction‐switching design further ensured that zebrafish larvae were loaded and captured in the trapping chamber with their heads forward in the microfluidic chip (Movie S2, Supporting Information). For quick larva detection, an algorithm was also developed in‐house to extract each frame from the real‐time recording and the head portion was then identified by binary image conversion and center of gravity detection as was demonstrated previously (Lin et al. Nature Communications).

### Preparation for Zebrafish Capsules

In order to rapidly immobilize nonanesthetized zebrafish larvae in a simple and highly parallel manner for multiphysiological imaging, a gelling platform was developed for high‐throughput preparing zebrafish capsules using agarose. After being automatically loaded into a microfluidic chip as mentioned above, 2% low gelling temperature agarose (which solidifies at ≈25 °C; A0701, Sigma) solution was injected into the chip at a rate of 13–15 mL/h^−1^ while its external temperature was controlled at ≈28 °C (Figure 2a). Then, the nonpermanently bonded PDMS layer was gently peeled off from the glass substrate after rapid gelation of agarose. And tens to hundreds of fish capsules with uniform length (6 mm, which ensured that the zebrafish larvae with ≈5 mm body length in 6–8 days after fertilization were completely embedded in the agarose and fully protected from any injury of cutting) were prepared by simple cutting and rinsed from the substrate into a container for further assessments.

### Preparation of Droplet Array Slide

An array with circular frosted areas (each had an inner diameter of 6 mm and an outer diameter of 7 mm) was engraved on a glass slide by a Ultraviolet (UV) laser in ≈5 min (laser marking speed: 30 mm/s^−1^; laser jump speed: 2000 mm/s^−1^; laser frequency: 50 kHz; laser energy: 80%). Superhydrophilic spots were formed by ≈4 min of plasma treatment. Following that superhydrophobic background was created by applying the superhydrophobic solution (MesoPhobic‐2000; MesoBioSystem) onto the patterned circle surface and air‐dried for 1 min. Based on the effect of discontinuous dewetting, aqueous solutions containing fish capsules or drugs were then rapidly separated on to the slide to form the microdroplet array (Figure S5, Supporting Information).

### Health and Behavioral Assessment

To assess the health status in larvae processed by the FC system, all larvae were immobilized in the agarose. Their health was monitored in the following 48 h. The criteria for health assessment include both functional and morphological abnormalities. Functional criteria included visual confirmation of normal heartbeat and blood flow rates. Morphological criteria included spine bending (i.e., lordosis, kyphosis, and scoliosis) and craniofacial abnormalities. For the behavioral test, all larvae were immobilized within capsules for 48 h and then released to assess their behaviors. The animals in the control group were freely moved in a culture dish. Then, the motor performance of the larvae upon 2 min light stimulation was recorded via a stereo microscope (SZ6060, Nanjing Jiangnan Novel Optics Co., Ltd.) equipped with a high‐speed camera (E31SPM20000KPA) for the manual analysis using ImageJ.

### Sandwiching Method for Parallel Compound Additions

For addition of compounds into the microdroplets containing zebrafish capsules, the sandwiching method reported recently was applied in this study.^[^ ^28^ ^]^ Initially, a fish plate with droplet arrays containing large‐scale FCs was prepared by discontinuous dewetting. Then a drug plate with droplets only containing the candidate drugs would be rapidly assembled in the same way. Using a motorized *X*–*Y*–*Z* stage, the drug plate would be precisely aligned to the fish plate that was fixed in the lower holder. After contraction with each other, the aligned drug droplets would release the drug by diffusion parallelly to the droplets containing FCs (Figure S10, Supporting Information). The strong capability to hold the droplets in the plate made the drug additions without any cross‐contamination.

### Concentration Validation

A micro‐spectrophotometer (K5800C, Beijing Kaiao Technology Development Co., Ltd.) was used to validate the final concentration of the solutions of CuSO_4_ based on the proportional relationship between the absorbance and the concentration (Figure S9, Supporting Information). Five samples with concentrations ranging from 0.04 to 0.64 mol L^−1^ were prepared for the determination of molar absorptivity of CuSO4 (Figure S9b, Supporting Information). This molar absorptivity was then used to determine the concentration of the diffused solutions in the FC system.

### Compound Library and Chemical Treatment

A small set (A set) of 16 neurological medicines and another set (B set) of drugs including 9 compounds with effects on the cardiovascular system were screened (Tables S1 and S2, Supporting Information). To validate the cardiovascular effects, each compound in B set was tested at concentrations of 100 × 10^−6^, 10 × 10^−6^, and 1 × 10^−6^ m. In the preliminary tests of the neuropharmacological effects, the concentrations of the drugs in set A also ranged from 1 × 10^−6^ to 100 × 10^−6^ m. All compounds were dissolved in dimethyl sulfoxide (DMSO) to prepare a stock solution of 10 × 10^−3^ m and subsequently diluted with E3 water to the final testing concentration. In the combinatorial drugs screen, the drugs in the B set were also tested with three concentrations (100 × 10^−6^, 10 × 10^−6^, and 1 × 10^−6^ m), while the concentration of clozapine and sertraline were set to 100 × 10^−6^ m based on the clustering results of their brain activity maps.

### Microscopy

Imaging was performed on a fully automated inverted fluorescent microscope (Leica THUNDER DMi8) equipped with a cooled high‐speed sCOMS camera (DFC 9000GT) and a 10× (NA, 0.4) objective. LAS X software was installed to control the microscope. When performing microscopic real‐time imaging on the larva to obtain data, the camera was operated at 10 Hz frame rate to ensure that no information was missed. To avoid evaporation of the droplet arrays in the FC system during long‐term imaging, a homemade wet chamber was adapted to the microscopic stage to keep a relatively humid environment.

### *T*‐Score BAM Generation from Calcium Imaging Data

Multiple procedures were introduced here to analyze the brain‐wide activities, which was similar with the previous demonstration.^[^ ^22^ ^]^ For each compound, the counts of calcium transients were obtained from five independent zebrafish larvae before and after compound treatment. To map the brain activities changes, each frame was first meshed into small regions of interest (ROIs) with a size of 15 µm^2^. The calcium transient counts (*c*) of each ROI were calculated by averaging the calcium signal changes

(1) $$c=\frac{1}{n}\sum_{i=1}^{n-1}\left(m_{i+1}-m_{i}\right)/m_{0}$$

where *n* represents the number of images and *m* is the pixel value of the calcium signal of each ROI.

By taking the changes (increase or decrease) of calcium transient counts in each ROI, a brain activity map (BAM) *M* = [*d_ij_*] was derived before (*t*_0_) and after chemical treatment (*t*_1_), and summed across multiple layers along the *Z*‐axis

(2) $$d_{ij}=\sum_{k}c_{ijk}^{t_{1}}-c_{ijk}^{t0}$$

where $c_{ijk}^{t_{0}}$ and $c_{ijk}^{t_{1}}$ are the calcium transient counts of the ROI at row *i* and column *j* of the *k*th layer before and after chemical treatment, respectively.

At last, a heat map describing a matrix of *T*‐scores (*T*‐score BAM), *T* = [*t_ij_*] was calculated for each ROIs across the five BAMs from different biological replicates by

(3) $$t_{ij}=\frac{\bar{d_{ij}}}{S_{d_{ij}}/\sqrt{n}}$$

where $\bar{d_{ij}}$ is the mean brain activity score of the ROI at row *i* and column *j* of the five BAMs, $S_{d_{ij}}$ is the standard deviation of the mean, and *n* is the number of biological replicates.

### Informatic Analysis of the *T*‐Score BAMs

Using the *T*‐score BAMs for the A set of neuroactive drugs, unsupervised classification was employed to dissect the phenotypic diversity. First, principal component analysis (PCA) was employed to reduce the dimensionality and noise. Then the consensus clustering based on hierarchical clustering was used to dissect the phenotypic diversity based on the top 10 principal components (Figure 4c). Four optimal phenotypic *T*‐score BAM clusters were identified, as the area under the empirical cumulative distributions’ functions curve did not increase substantially (<1%) from 4 to 5 clusters.

To associate the identified phenotypic *T*‐score BAM clusters with therapeutic drug categories, hypergeometric tests were performed for overrepresentation. In these tests, the hypergeometric *p*‐value was calculated as the probability of observing *k* or more drugs of an ATC category in total *n* drugs of a specific *T*‐score BAM cluster. The significant overlap (*p* < 0.05) between a *T*‐score BAM cluster and an ATC category was regarded as the signature subgroup. For example, Class 1, Class 4 showed significant association with N06, N05, respectively (Figure 4d,e).

### Heartbeat Analysis

In order to eliminate the individual differences among the animals, the cardiac signals were recorded before and after different drug treatments in each larva. Using the software ImageJ, the heartbeat signals were manually extracted via calculating the changes of pixel values in the ROI (Figure 5a) resulting from the contraction–relaxation cycle of the heartbeat (Figure S13, Supporting Information). Matlab was then utilized to calculate the heart rate and plot the corresponding heart rate variation curve. To statistically analyze the long‐term cardiac effects of drugs, a fluctuation index (heart beat fluctuation index, HBFI) was defined to represent the heart rate modulations of each drug

(4) $$\mathrm{HBFI}=\sum_{i=1}^{T}\left[\left(r_{i+1}-r_{i}\right)/r_{i}\right]$$

where *r_i_* is the heart rate of the animal in the *i*th minute and *T* is the recording time.

In the further cardiac analysis, the envelopes and peaks were extracted from the raw data using specific functions in Matlab (Figure S15, Supporting Information). The low frequency (<0.2 Hz) noise was filtered by a Highpass Butterworth Filter using the “butter” function. The peaks of the signal were extracted by detecting peak‐to‐peak values. In this study, the peak‐to‐peak values were calculated by correspondently subtracting the trough value vector from the peak value vector in Matlab. To analyze the signals in the frequency domain, the fast Fourier transform was then performed to derive the spectrum information. The sample code was detailed in Table S3 (Supporting Information).

### Microinjection

A microinjector (PL1‐100A, Warner Instruments) coupled with a micromanipulator (MN‐153, Narishige), a stereo microscope (Leica S9E) and a micropipette (fabricated via a puller, PC‐100, Narishige) was used to perform microinjection in the oriented and immobilized animals in the capsules (Figure S19, Supporting Information). The fluorescein isothiocyanate (FITC) dye (D‐9801, Bioss) was then delivered to the targeted regions.

### Statistical Analysis

All experiments were repeated at least five times. The data obtained from this study were used without preprocessing unless noted otherwise. The bar and curve graph data were presented as the mean ± standard error of mean (s.e.m.). For the box plots, the vertical centerline indicated the median, while the width of the box and error bar represented the interquartile range (IQR) and 1.5 times the IQR, respectively. Statistical analysis was performed by one‐way analysis of variance (ANOVA) using SPSS Statistics 25 (IBM Corp., Armonk, NY). Tukey's post hoc test was used for multiple post hoc comparisons to determine the significance between the groups after one‐way ANOVA. The difference between groups was considered statistically significant for ^*^ *p* < 0.05 and very significant for ^**^ *p* < 0.01.

## Conflict of Interest

The authors declare no conflict of interest.

## Author Contributions

M.T. and X.D. contributed equally to this work. X.L. conceived and coordinated the project. P.S., H.L., and X.L. designed the research. M.T. designed and fabricated the FC system, performed the high‐throughput drug screening, and analyzed the data. X.D. developed the computational tools and performed the informatic analysis. S.H., A.Y., and L.Z contributed to data processing. Y.Z., Y.L., and L.X. helped with zebrafish maintenance. M.T. and X.L. wrote the manuscript. P.S. and H.L. also contributed to the writing of the manuscript.

## Supporting information

Supporting Information

Supplemental Movie 1

Supplemental Movie 2

Supplemental Movie 3

Supplemental Movie 4

Supplemental Movie 5

Supplemental Movie 6

Supplemental Movie 7

## Data Availability

The data that support the findings of this study are available from the corresponding author upon reasonable request.
