# Supplementary material for: Fish Capsules: A System for High‐Throughput Screening of Combinatorial Drugs
Source: Adv Sci (Weinh). 2022 Jan 27;9(9):2104449. doi: 10.1002/advs.202104449 (PMC8948576; doi:10.1002/advs.202104449)
Supplement: Supplementary file 1 — Supporting Information [file ADVS-9-2104449-s006.pdf]

ADVANCED SCIENCE
Open Access

Supporting Information

for *Adv. Sci.*, DOI 10.1002/advs.202104449

Fish Capsules: A System for High-Throughput Screening of Combinatorial Drugs

Minghui Tang, Xin Duan, Anqi Yang, Shijie He, Yajing Zhou, Yuxin Liu, Lu Zhang, Xuan Luo, Peng Shi, Honglin Li* and Xudong Lin**

ADVANCED SCIENCE
Open Access

Supporting Information

for *Adv. Sci.*, DOI: 10.1002/advs.202104449

Fish capsules: a system for high-throughput screening of combinatorial drugs

Minghui Tang, Xin Duan, Anqi Yang, Shijie He,Yajing Zhou, Yuxin Liu, Lu Zhang, Xuan Luo, Peng Shi, Hong lin Li*, Xudong Lin**

Supporting Information
Fish capsules: a system for high-throughput screening of combinatorial drugs

Minghui Tang, Xin Duan, Anqi Yang, Shijie He,Yajing Zhou, Yuxin Liu, Lu Zhang, Xuan Luo, Peng Shi*, Hong lin Li*, Xudong Lin*

M. Tang, X. Duan, A. Yang, S. He, Y. Zhou, Y. Liu, L. Zhang, Dr. X. Lin
Guangdong Provincial Key Laboratory of Sensor Technology and Biomedical Instrument
School of Biomedical Engineering
Sun Yat-Sen University,
Guangzhou, China
E-mail: linxd37@mail.sysu.edu.cn

X. Luo, Prof. P. Shi
Department of Biomedical Engineering
City University of Hong Kong
Kowloon, Hong Kong SAR, China
Shenzhen Research Institute
City University of Hong Kong
Shenzhen, Guangdong, China
E-mail: pengshi@cityu.edu.hk

Prof. H. Li
State Key Laboratory of Bioreactor Engineering, Shanghai Key Laboratory of New Drug Design
School of Pharmacy
East China University of Science and Technology,
Shanghai, China
E-mail: hlli@ecust.edu.cn

Supplementary Figures

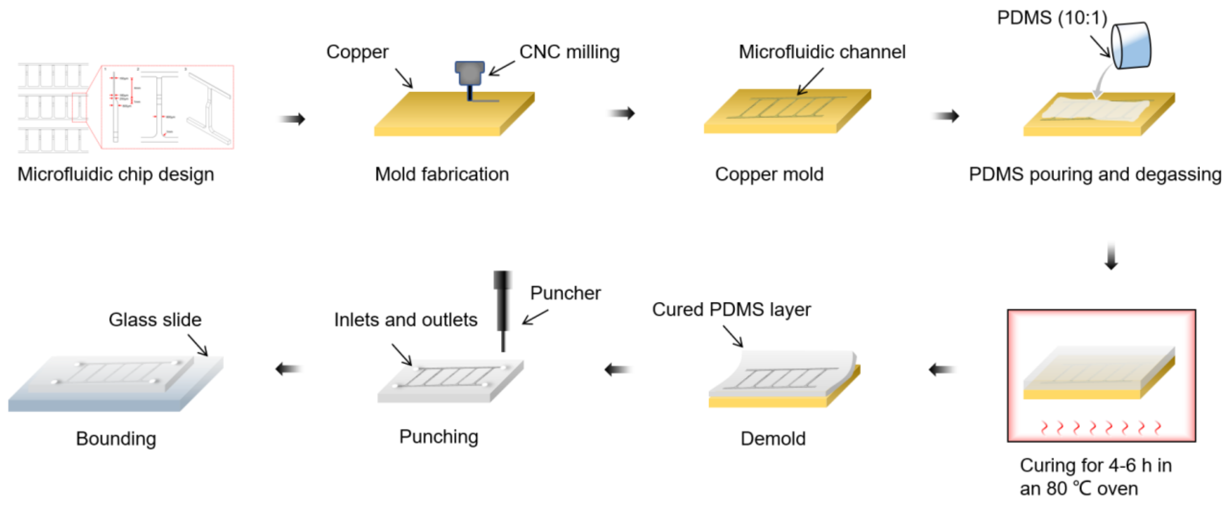

Figure S1. Fabrication of microfluidic devices based on computer numerical control (CNC) machining. First, a positive mold of the microfluidic design was machined on a plain copper substrate with 30 μm resolution. After soaking in 100% alcohol overnight, the mold was rinsed with deionized water a couple of times and dried in the air. Then polydimethylsiloxane (PDMS) was used to make the chips with flow channels by replicating molding from the copper molds. PDMS was poured onto the mold and baked in an 80 °C oven for 4-6 hours after completely removing the air bubbles in a vacuum chamber. Then the cured PDMS layer was demolded for further punching to form the inlets and outlets. Last, the modified PDMS layer was temporarily bonded to the glass substrate to make the final microfluidic chip that used in our FC system.

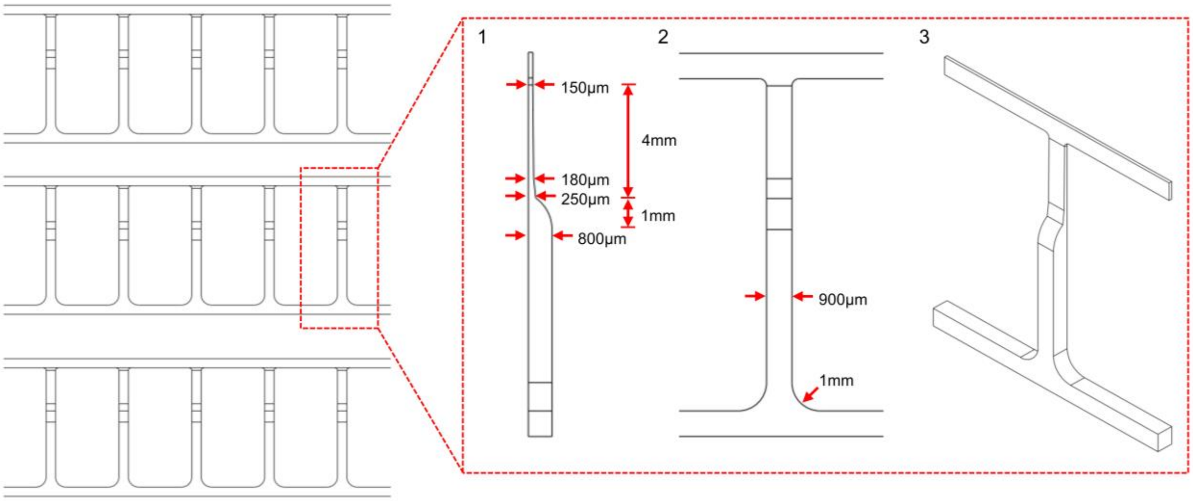

Figure S2. Detailed design of the microfluidic chip array for zebrafish manipulation. Insets illustrations show the side view (1), top view (2) and 3D view (3) of the chip respectively.

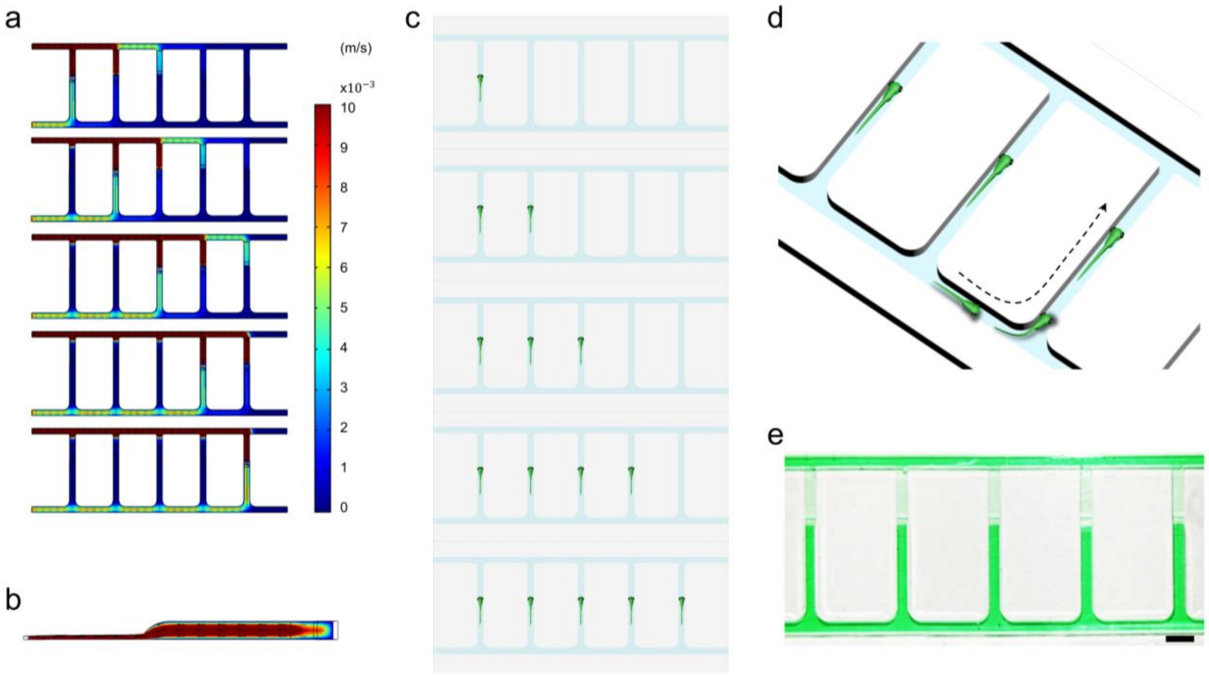

Figure S3. Automated zebrafish loading, orientation and immobilization based on hydrodynamic force. (**a-b**) Simulation results of the flow dynamics within a chip before, during and after larva loading, indicating a hydrodynamic force pointing towards the trapping chambers one after another. (**a**) Top view of the simulation results: from top to bottom represents no larva trapped, one larva trapped, two larvae trapped, three larvae trapped and four larvae trapped respectively. (**b**) Side view of the simulation results in a trapping chamber. (**c-d**) Illustrations showing the automated manipulation of zebrafish larvae with their heads forward. (**e**) A representative image of the microfluidic device. Scale bar, 2 mm.

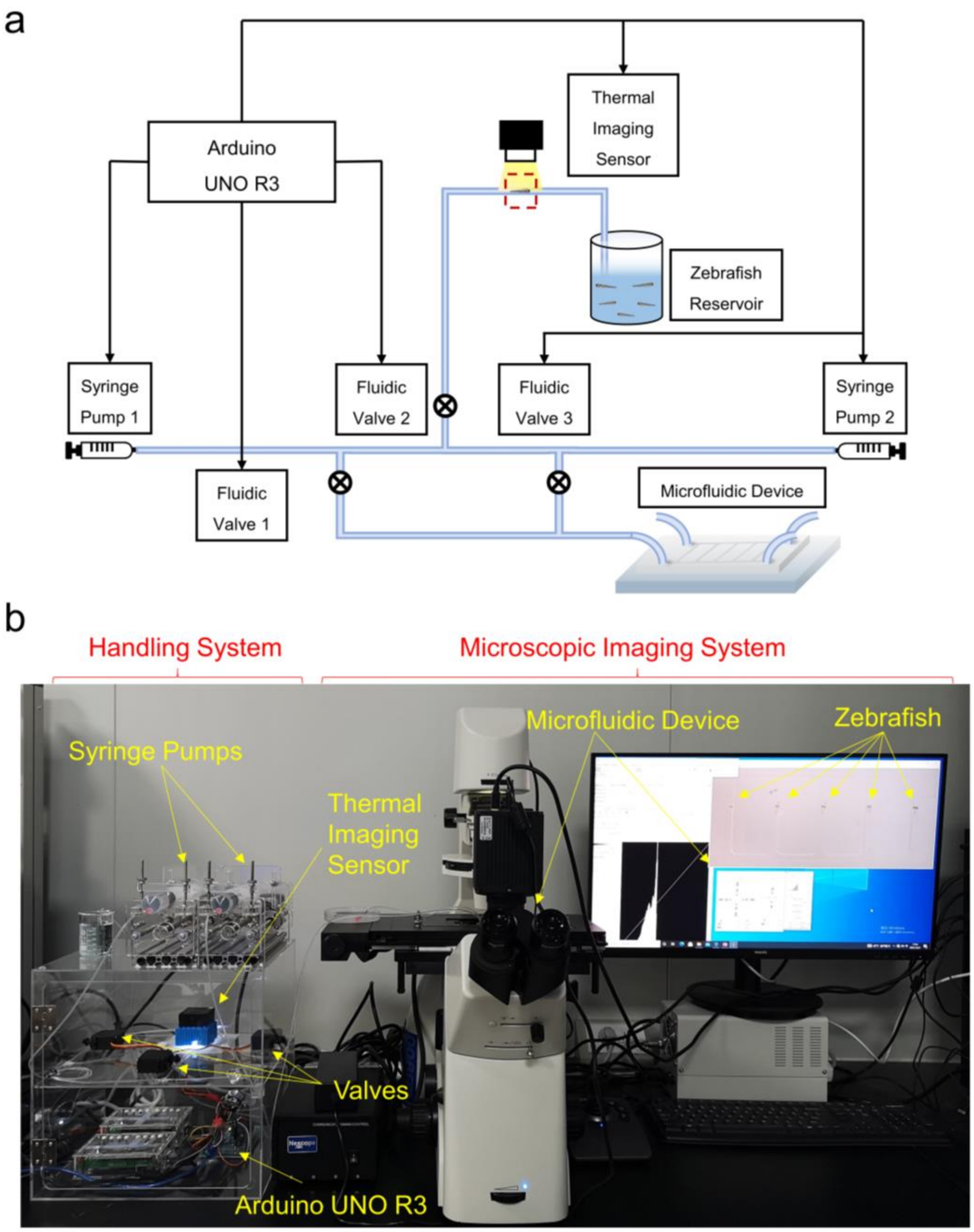

Figure S4. A homemade system for handling larval zebrafish. Schematic (**a**) and image (**b**) of the automated system for the manipulation of zebrafish larvae. Briefly, an Arduino board (Arduino UNO R3, a microcontroller board based on the ATmega328P) was applied here to digitally control the pumps and the electromagnetic fluidic valves. Two syringe pumps were used to load the zebrafish larvae from a reservoir into the fluidic circuitry. A sensing box based on thermal imaging sensors was developed to detect the fish loading and the direction of the animal head, which was then used as the trigger signal to actuate the corresponding pumps and valves. This automatic direction-switching design further ensured that zebrafish larvae were loaded and captured in the trapping chamber with their heads forward in the microfluidic device.

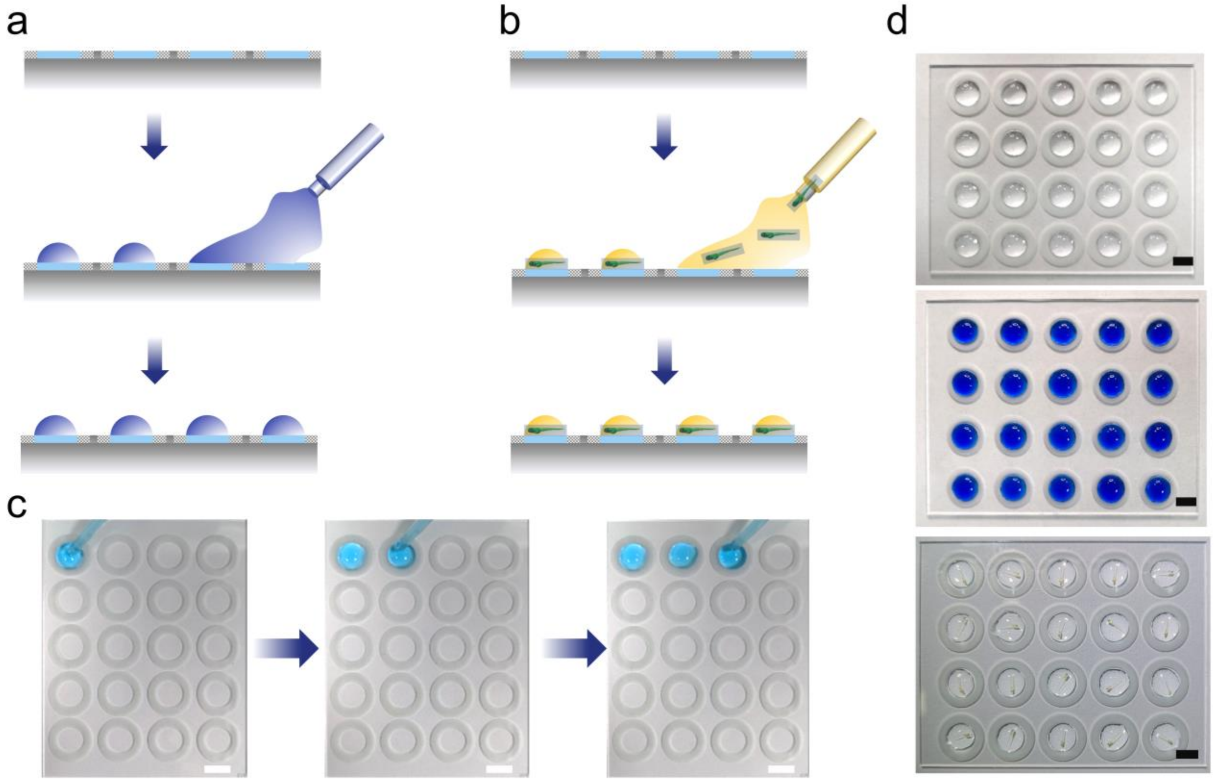

Figure S5. Preparation of a micro-droplet array based on the effect of discontinuous dewetting. **(a-b)** Schematic representation of micro-droplet generation (**a**) and deposition of zebrafish capsules into the microdroplet array **(b)**. **(c)** Images of the process of spreading aqueous solution using the effect of discontinuous dewetting. Scale bar, 7mm. **(d)** Images exhibited micro-droplet array containing water solution (top), drugs (middle) and fish-capsules (bottom). Scale bar, 5 mm.

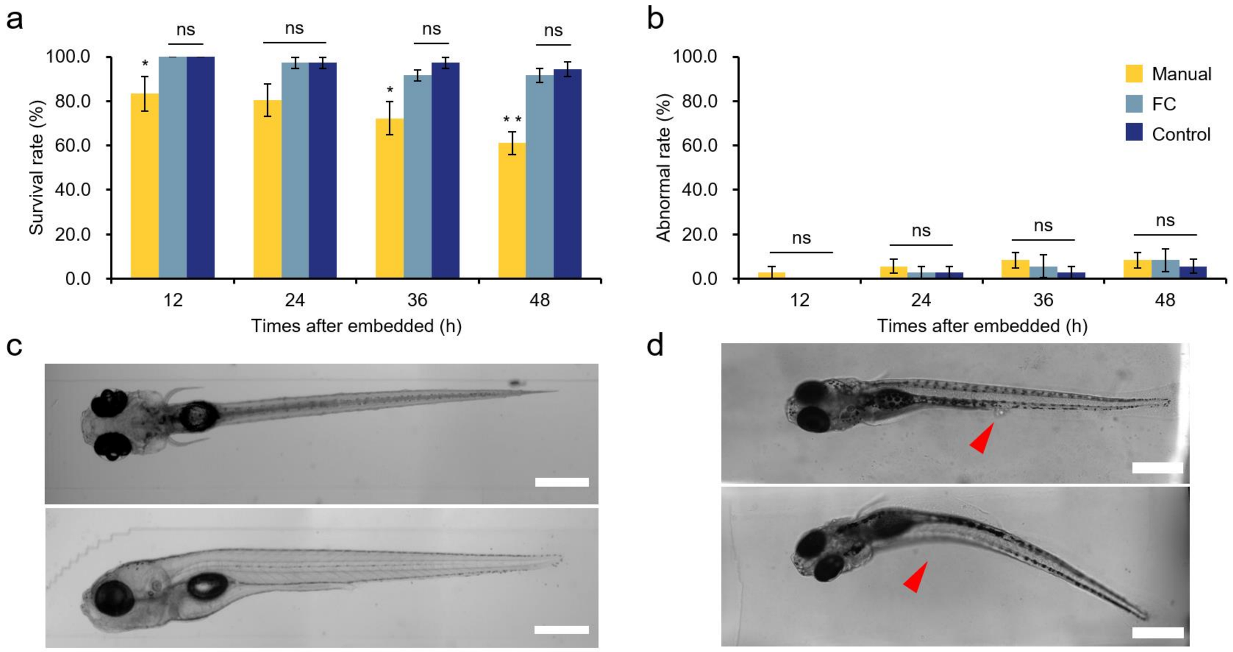

Figure S6. Health assessment of larvae immobilized by the FC system and manual manipulation, respectively. (**a-b**) Survival rate **(a)** and abnormal rate **(b)** of zebrafish larvae in a consecutive 48 hours period. Total of 36 larvae were evaluated from six independent experiments (6 larvae/experiment). Error bars indicated standard error of mean (s.e.m); "ns" indicated no significant difference, *p<0.05, **p<0.01 by one-way analysis of variance (ANOVA). **(c-d)** Images showed the apparent comparison between zebrafish capsules **(c)** and manual encapsulation **(d).** Red marks indicated spinal curvature or physical injury. Scale bar, 0.5 mm.

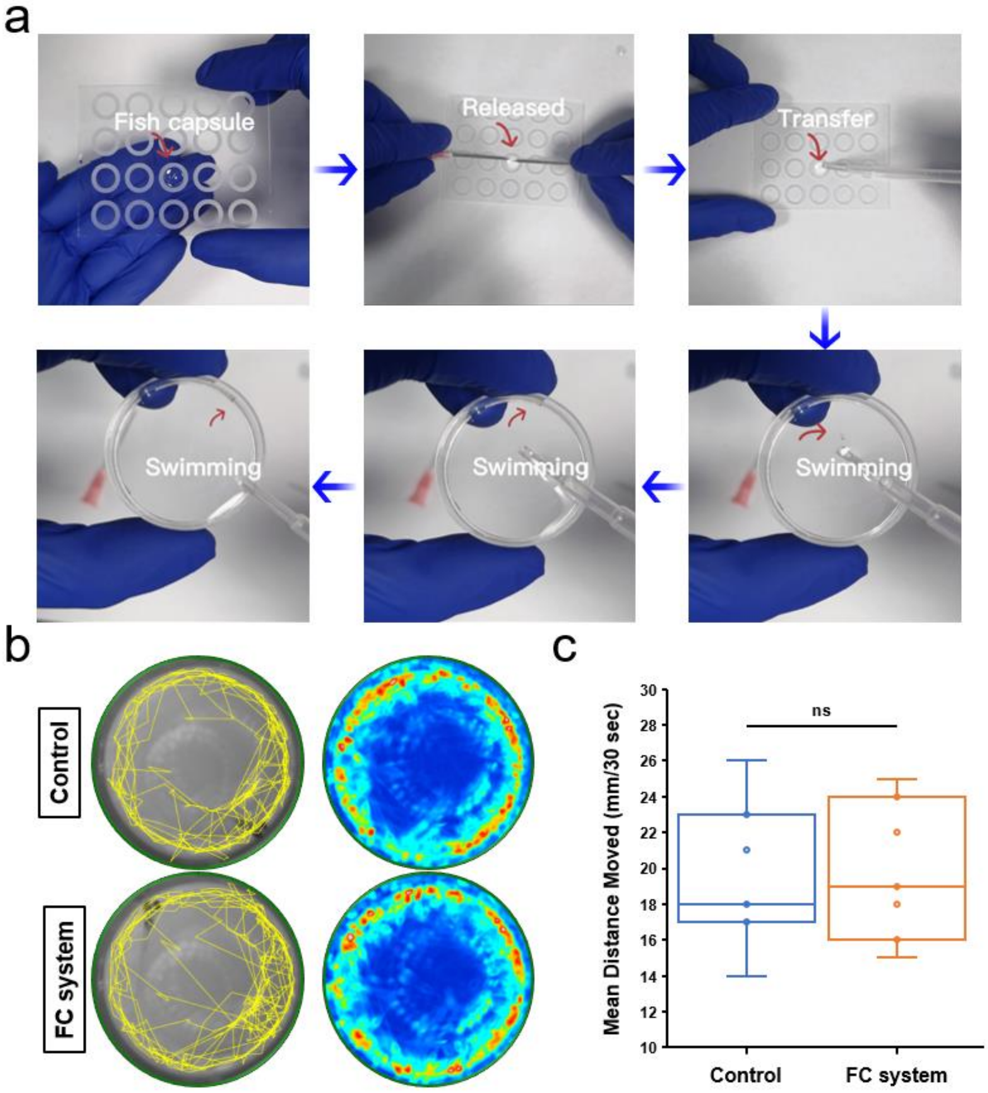

Figure S7. Behavioral test of the larvae released from the FC system. (**a**) Images showing the release of a larva from the capsule. **(b)** The representative track maps of the general behaviors and heat maps of time spent in the culture dishes of the zebrafish larvae. **(c)** The statistical analysis of locomotive behaviors in the animals released from the FC system, compared to the controls (freely moving in the culture dish). Larval behavior and movement were monitored for 15 minutes after a 2 minute light stimulation. n = 12; “ns” indicated no significant difference by one-way analysis of variance (ANOVA).

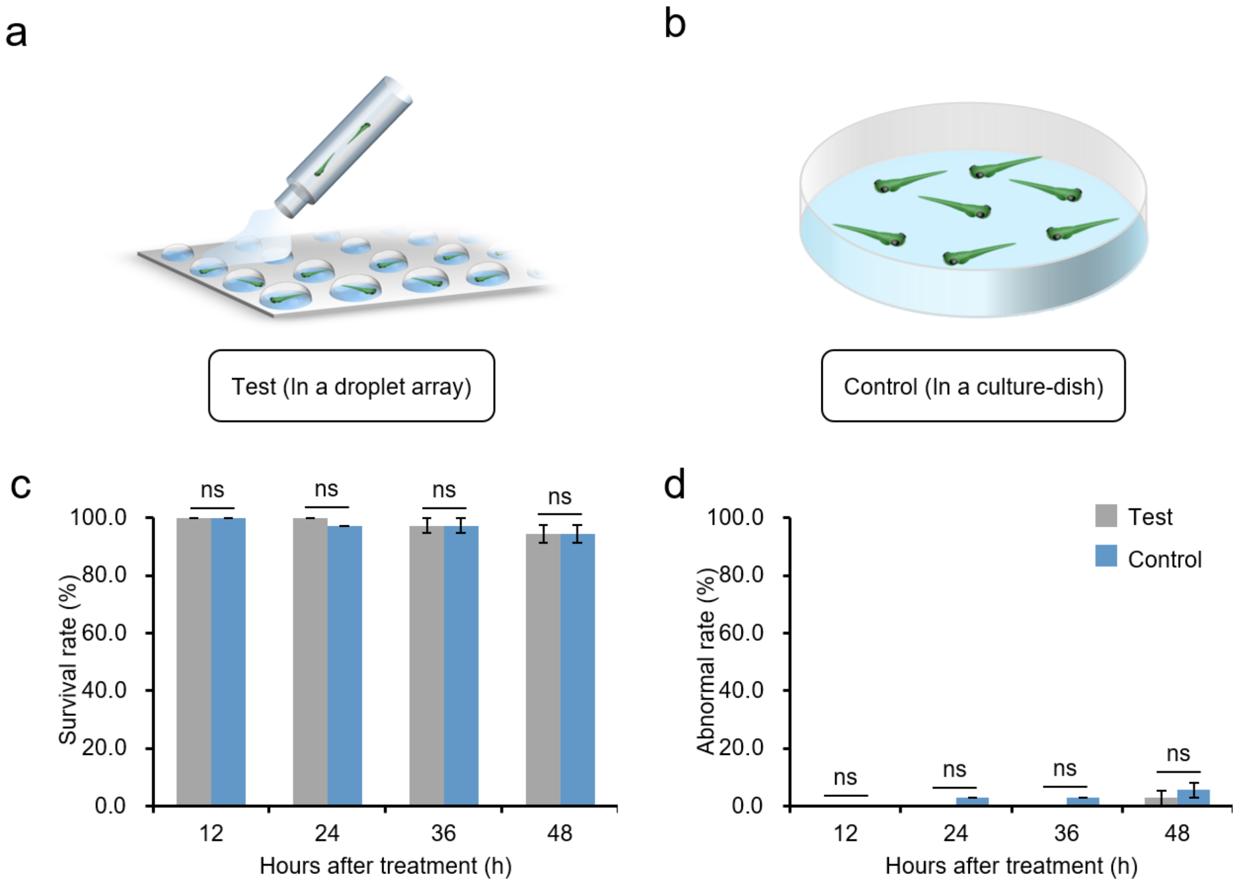

Figure S8. Toxicity validation of superhydrophobic coating in the droplet array. (**a-b**) Zebrafish larvae were incubated in droplet array (**a**) and a culture-dish (**b**). (**c-d**) Survival rate (**c**) and abnormal rate (**d**) of zebrafish larvae in test group (in a droplet array) and control group (in a culture dish) during a consecutive 48 hours period. Total of 36 larvae were evaluated from six independent experiments (6 larvae/experiment). Error bars indicated standard error of mean (s.e.m); "ns" indicated no significant difference by one-way analysis of variance (ANOVA).

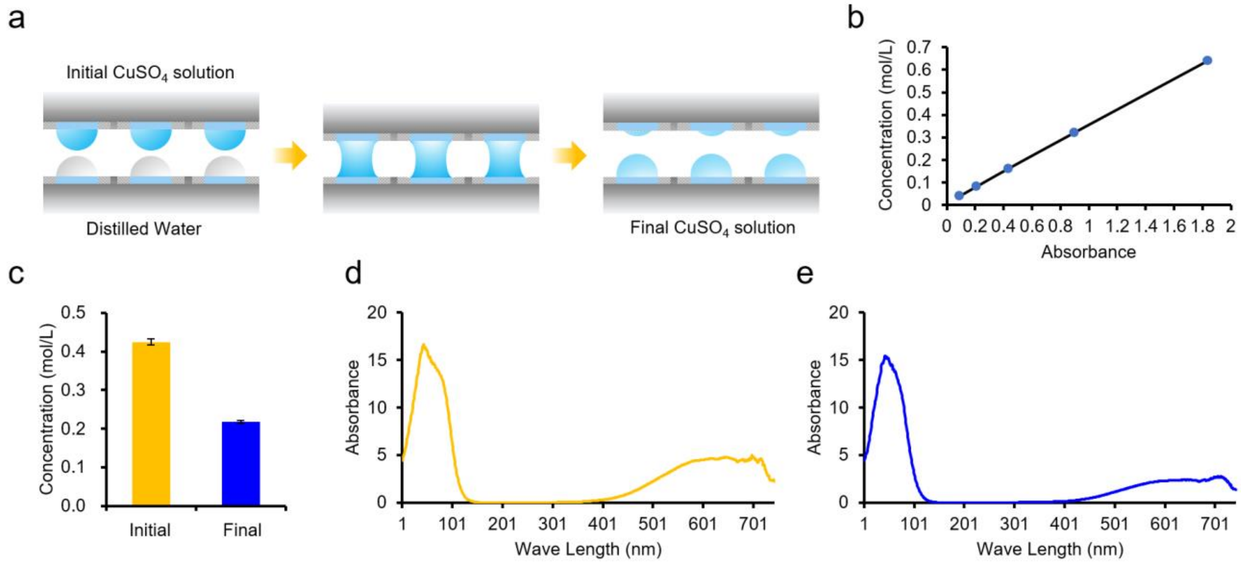

Figure S9. Concentration validation using a micro-spectrophotometer. (**a**) Schematic showing the parallel compound addition of $CuSO_4$ solutions via sandwiching method. (**b**) Determination of molar absorptivity of $CuSO_4$ based on the detection wavelength at 650 nm; five standard solutions with different concentrations (0.04, 0.08, 0.016, 0.32, 0.64 mol/L) were applied. Error bars indicated standard error of the mean (s.e.m.), n = 3. (**c-e**) Comparisons of the concentration (**c**) and absorbance (**d**, initial solutions; **e**, final solutions) between the initial solution and the final solution after 1-minute incubation and transfer in the FC system. Error bars indicated standard error of mean (s.e.m); n = 6.

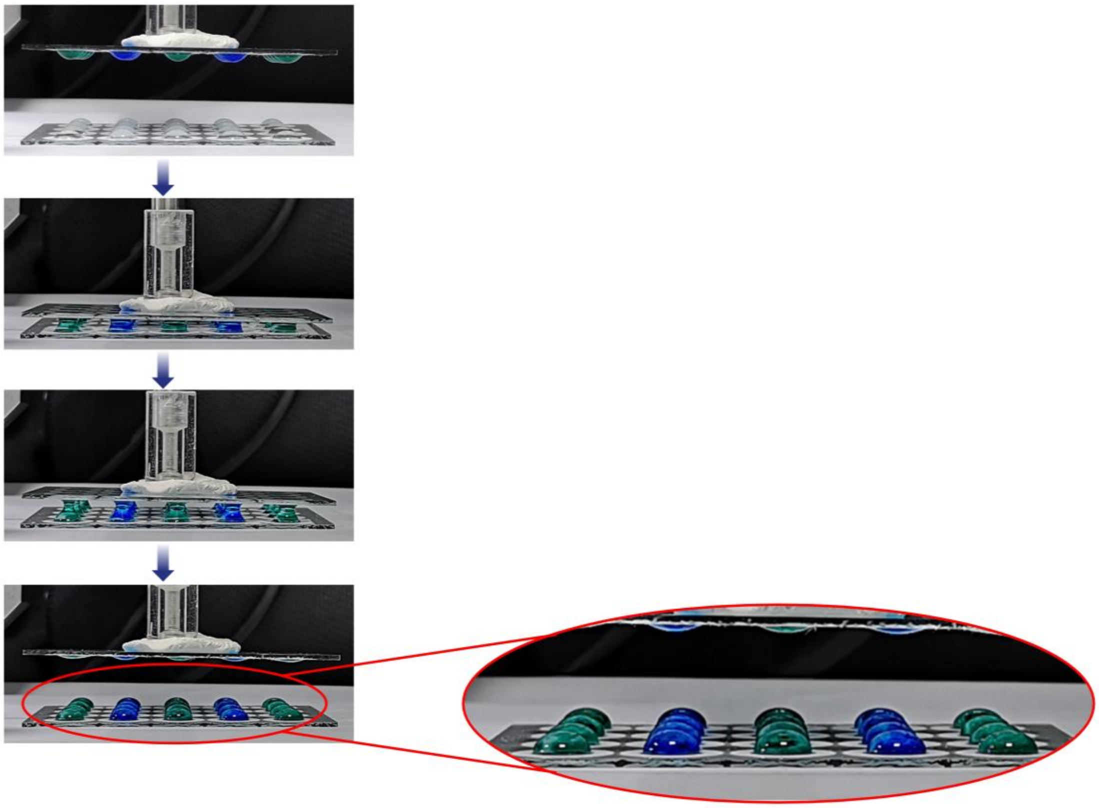

Figure S10. Images showed the parallel compound addition using sandwiching method. No cross-contamination was induced during parallel compound addition.

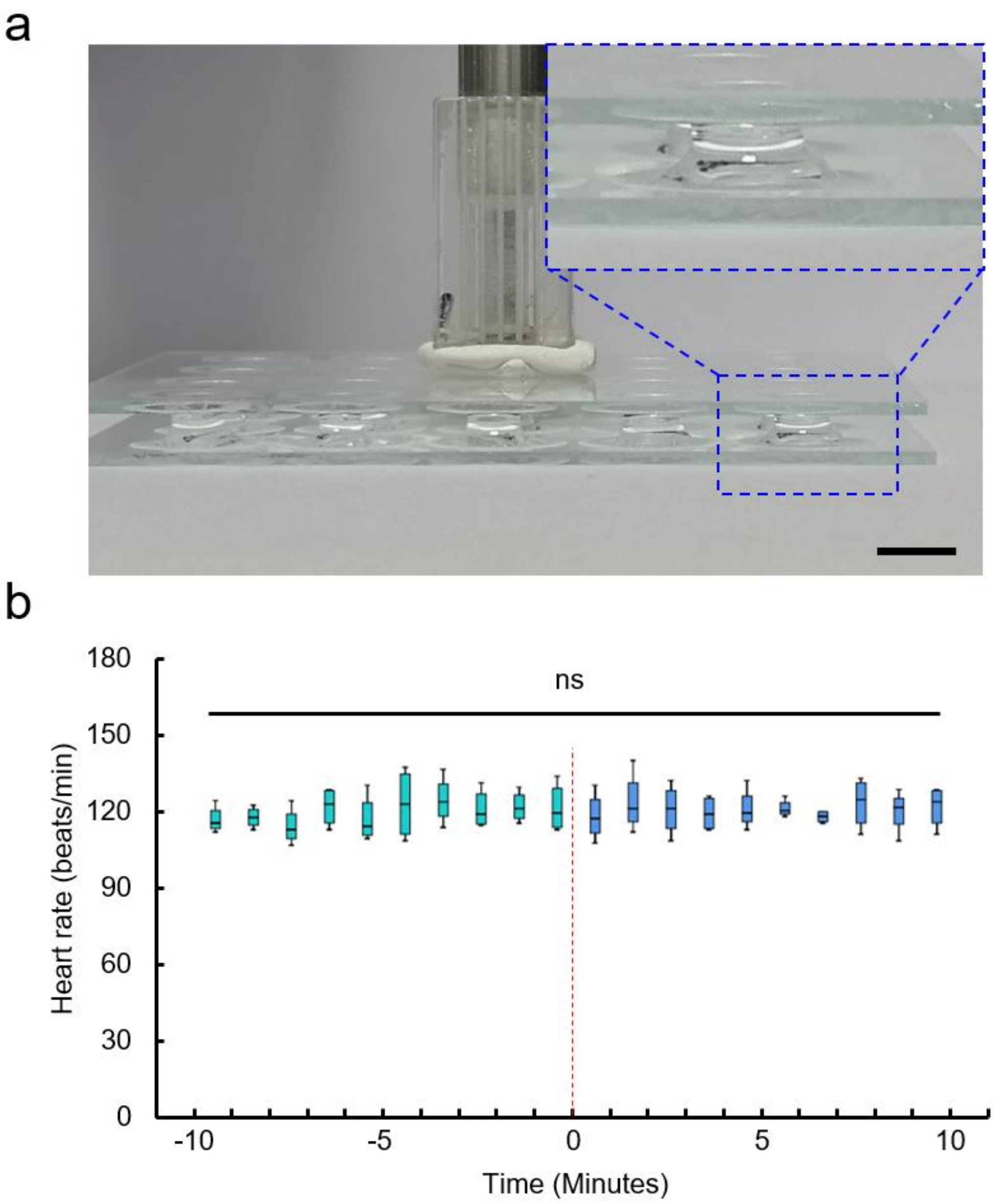

Figure S11. Cardiac function monitoring during parallel compound addition using sandwiching method. (**a**) Images showing the parallel addition of E3 water using the sandwiching method for further physiological evaluation. For clarity, the image of the blue-boxed regions is enlarged. Scale bar, 6 mm. (**b**) Quantification of the heart rates of larvae before and after water addition, indicated that no stress was induced during parallel addition via sandwiching strategy. The starting point of the parallel addition was set to be “0 min” and indicated by the red dotted line. n = 4; “ns” indicated no significant difference by one-way analysis of variance (ANOVA).

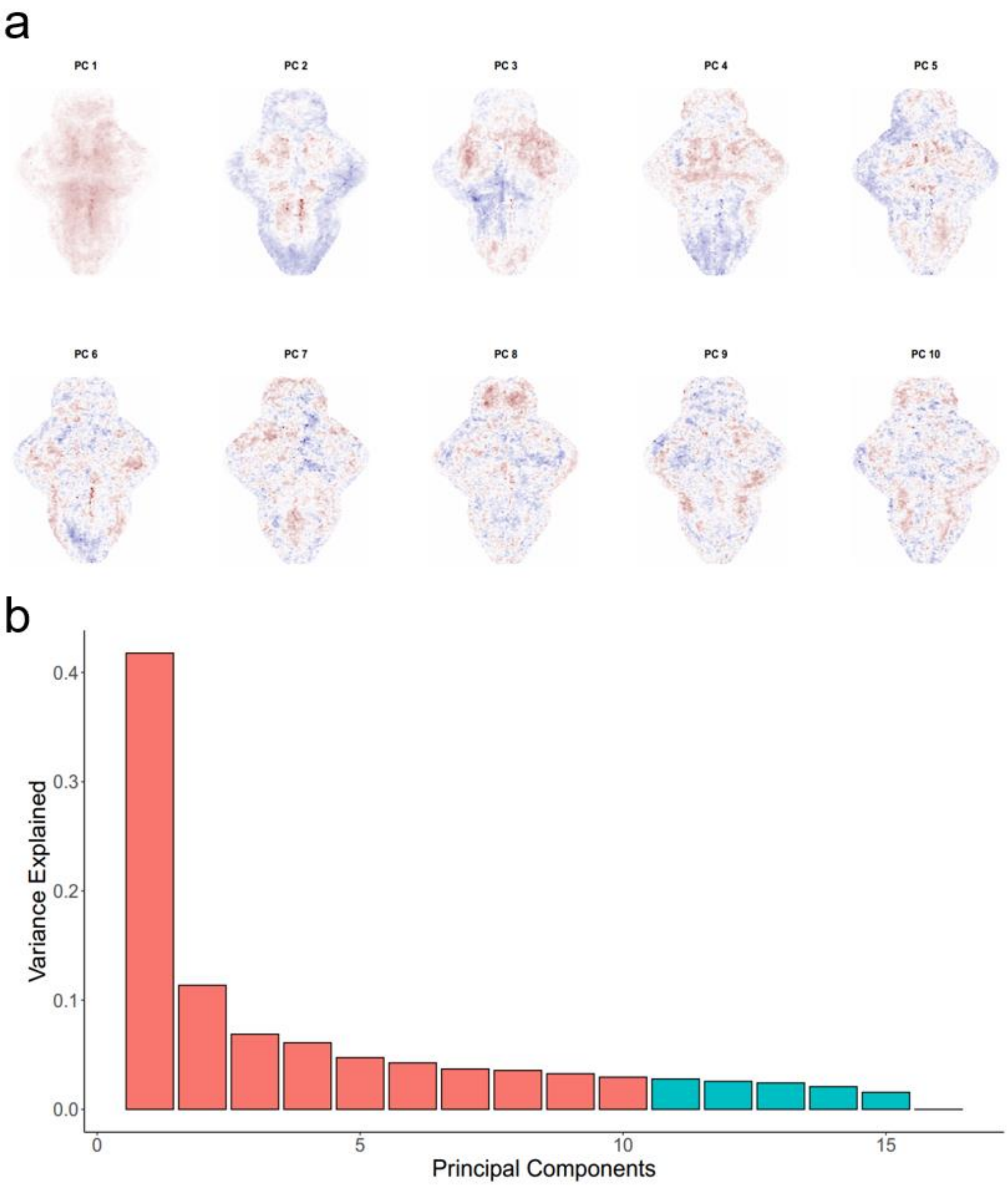

Figure S12. The top 10 PCs account for the major variation of T-score BAMs. (**a**) Images of the pheno-print vectors for the top 10 PCs; (**b**) top 10 PCs account for 89% total variance.

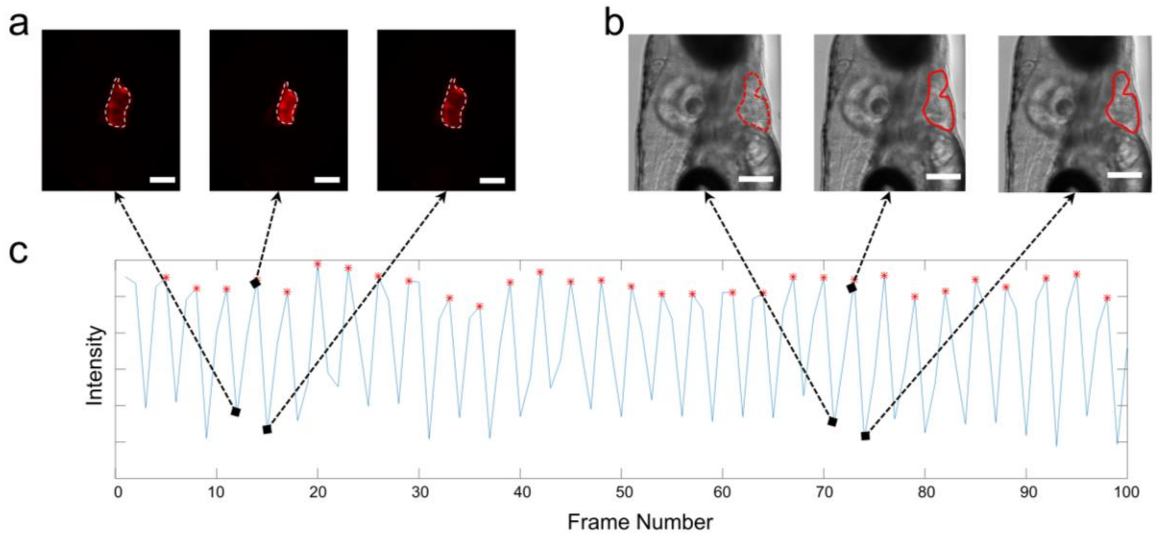

Figure S13. The measurement of the heartbeats. Analysis of heartbeats by measuring the volume change of the heart during a contraction-relaxation cycle in both transgenic **(a)** and wild-type **(b)** zebrafish. Scale bar, 100 µm. **(c)** Heartbeat signals were then extracted from the microscopic recording via calculating the changes of pixel values in the ROI using the software ImageJ and Matlab was then utilized to find the peaks and calculate the heart rate.

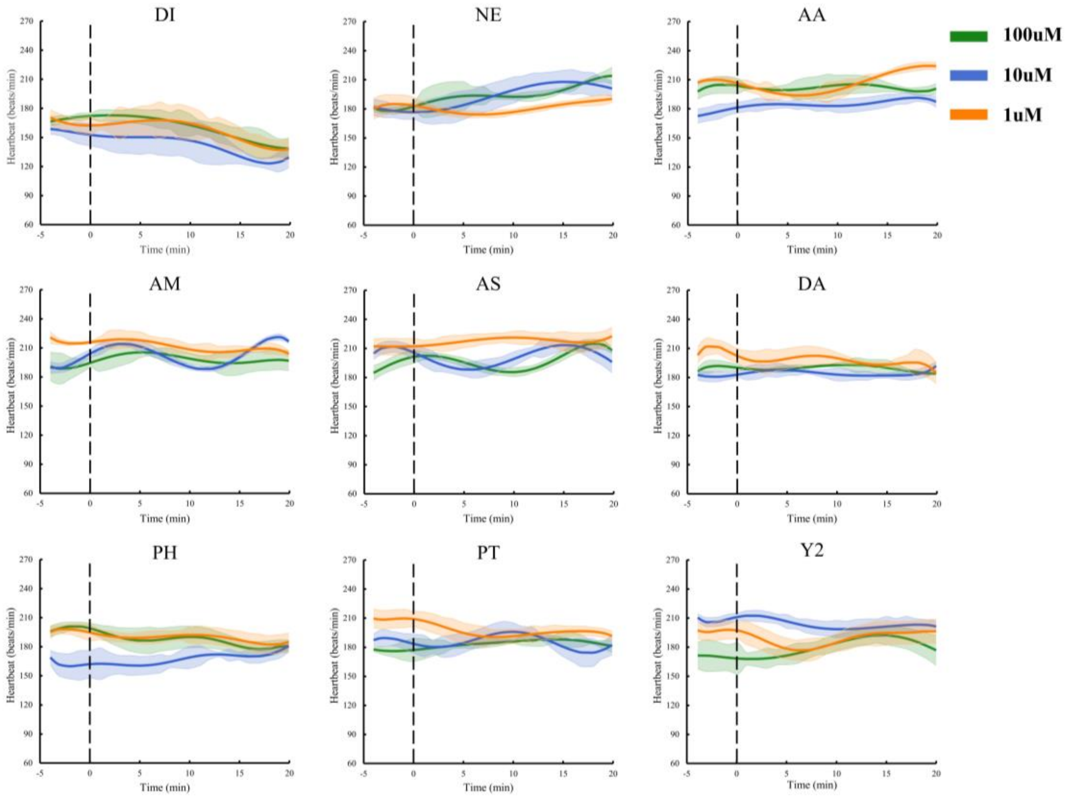

Figure S14. Heart rate fluctuations in the mono-therapy screen. Cardiac effects induced by 9 compounds including Disulfiram (DI), Norepinephrine (NE), Aminobutyric acid (AA), Amitriptyline (AM), Aspirin (AS), Dopamine (DA), Phenacetin (PH), Phenytoin (PT) and Y-27632 (Y2) with different concentrations on zebrafish larvae. The dotted line indicated the drug treatment. The shade indicated s.e.m.

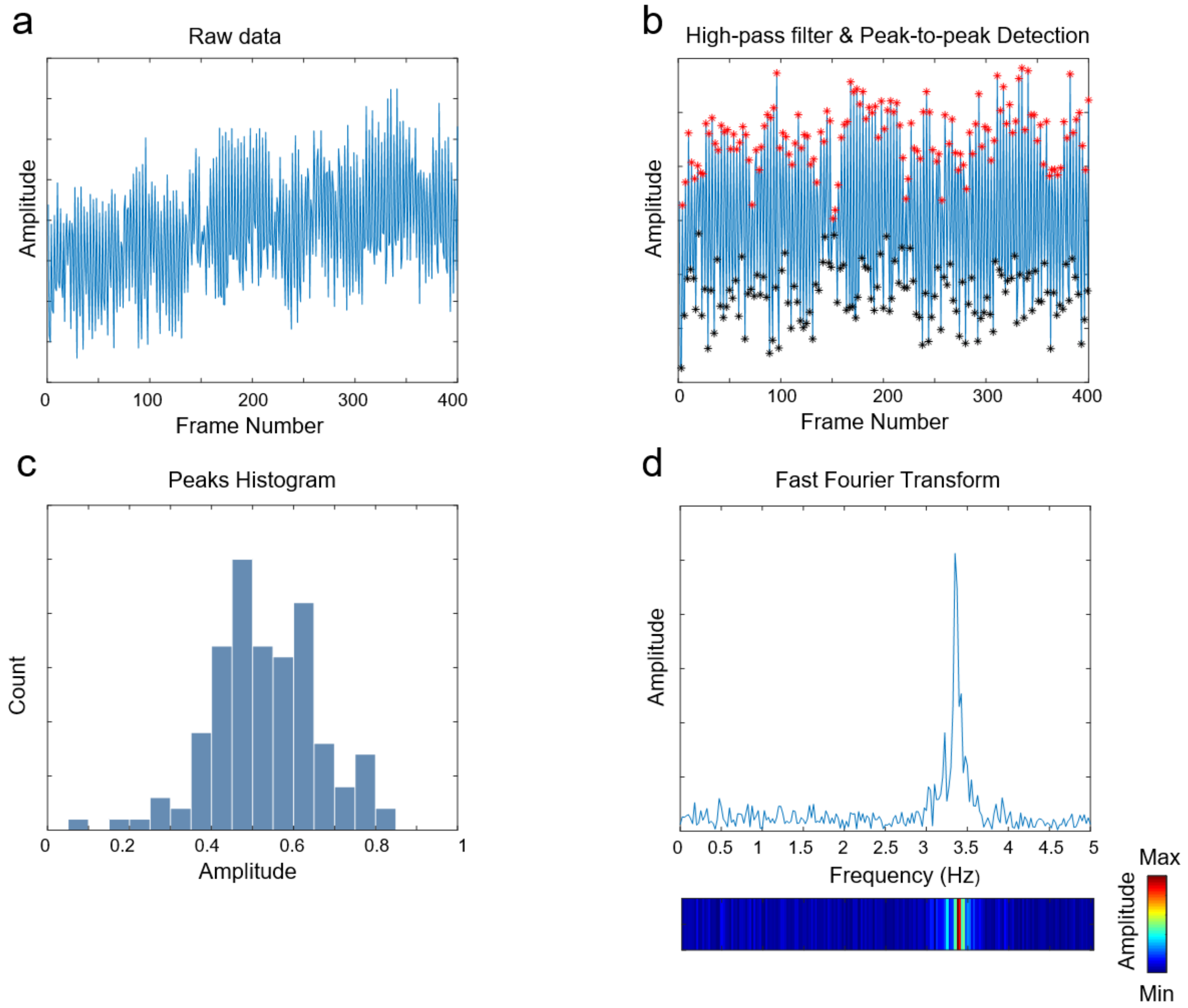

Figure S15. The procedures of heartbeat signal analysis. (a) Raw data from the original recording. **(b)** High-pass filter and peak-to-peak detection of the heartbeat signal. **(c)** Peaks histogram from **(b)**. **(d)** Spectrum pattern from the fast Fourier transform (FFT) showed single-side amplitude spectrum of heartbeat signal.

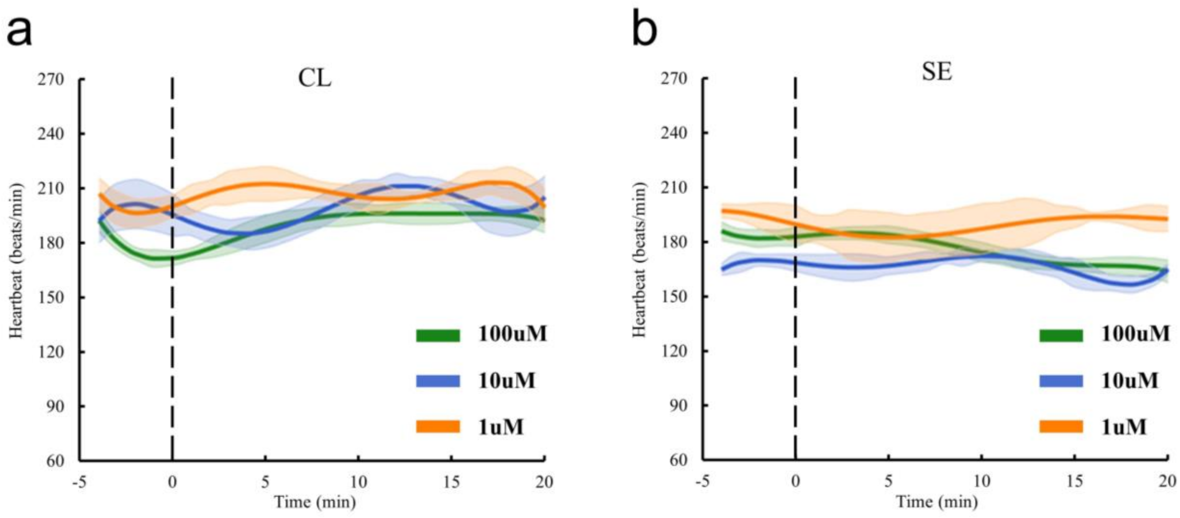

Figure S16. Heart rate fluctuations of clozapine and sertraline. Dynamic effects of heart beat under clozapine (**a**) and sertraline (**b**) treatment at doses from 1 µM to 100 µM. The dotted line indicated the drug treatment. The shade indicated s.e.m.

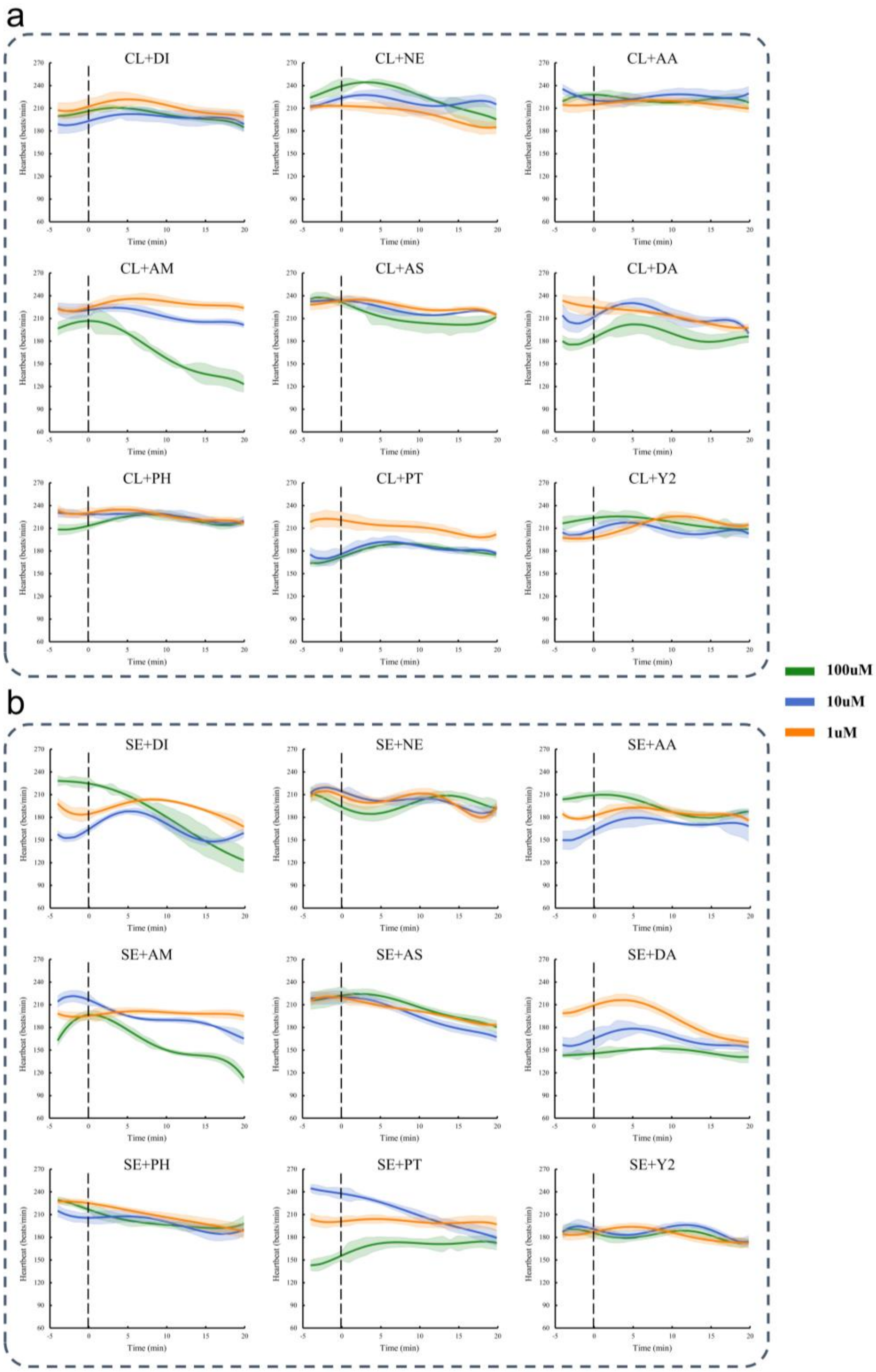

Figure S17. Heart rate fluctuations in the poly-therapy screen. **(a)** Heartbeat curves under combined treatment of 100 μM clozapine and different concentrations of 9 compounds. **(b)** Heartbeat curves under combined treatment of 100 μM sertraline and different concentrations of 9 compounds. The dotted line indicated the drug treatment. The shade indicated s.e.m.

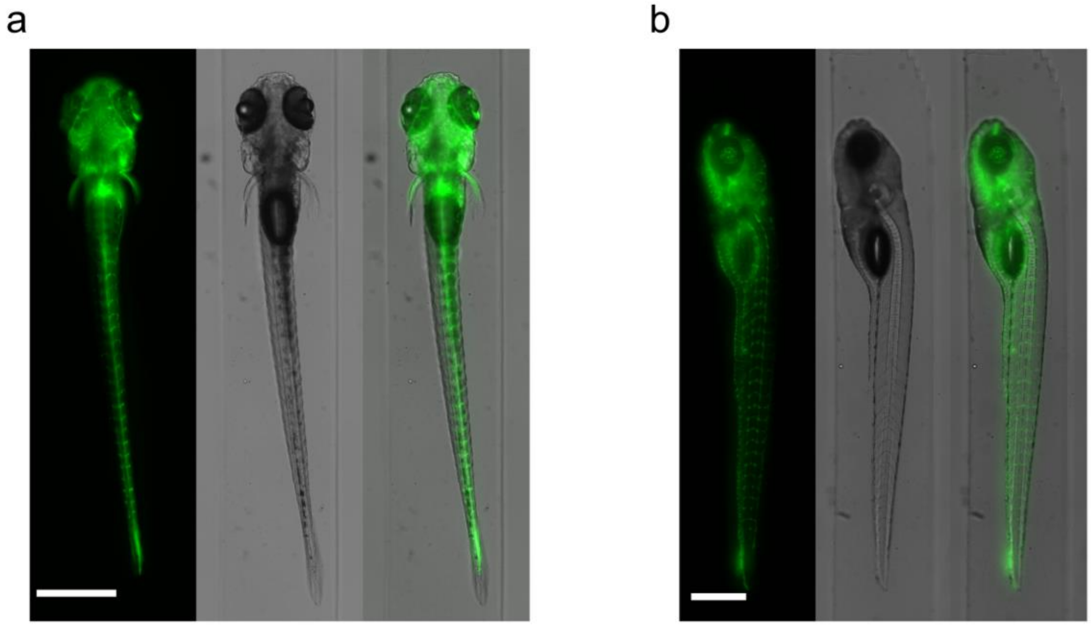

Figure S18. Representative images of the Kdrl1:eGFP transgenic larvae from in the FC system. (**a-b**) white-field and fluorescent images with the dorsal-up orientation (**a**) and the lateral orientation (**b**). Scale bar, 0.5 mm.

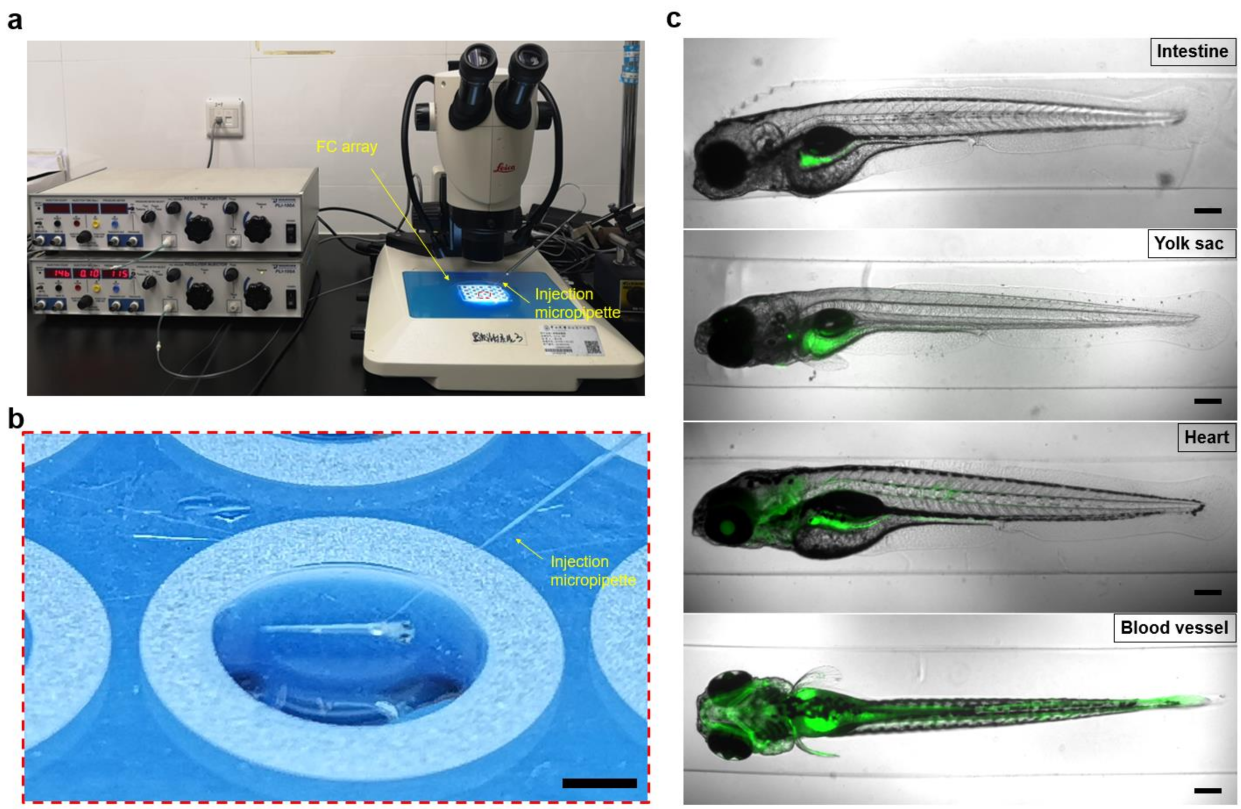

Figure S19. Microinjection based on the FC system. (**a**) Setup of the system. Oriented larvae in the agarose-based capsules were arrayed in the microdroplets. An injection micropipette was utilized to deliver the fluorescein isothiocyanate (FITC) dye to the targeted regions of the animal. (**b**) The enlarged image of the boxed regions in figure (**a**); scale bar, 1 mm. (**c**) Images of zebrafish larvae following microinjection of FITC-coupled dye into different organs based on FC system. Scale bar, 200 μm.

Supplementary Tables

Table S1. List of drugs abbreviation and function of Set A

NO.	Drug name	Abbr.	Function*
1	Clozapine	CL	A diazepine antiepileptic drug, is commonly accompanied by side effects such as tachycardia, myocarditis and cardiomyopathy[1, 2].
2	Sertraline	SE	An antidepressant, is a selective serotonin reuptake inhibitor (SSRI) usually resulting in bradycardia[3].
3	Fluoxetine	FL	An antidepressant, is a selective serotonin reuptake inhibitor used to treat major depressive disorder, bulimia, OCD, premenstrual dysphoric disorder and panic disorder[4].
4	Tranylcypromine	TR	A monoamine oxidase inhibitor used to treat major depressive disorder[5].
5	Oxcarbazepine	OX	An ion channel modulator, is an anti-epileptic used in the treatment of partial-onset seizures[6].
6	Perampanel	PE	A non-competitive AMPA modulator, used to treat partial-onset seizures with or without secondarily generalized seizures[7].
7	Carbamazepine	CA	An anticonvulsant, used to treat various types of seizures and pain resulting from trigeminal neuralgia[6].
8	Chlorprothixene	CH	A thioxanthene antipsychotic, used to treat psychotic disorders and of acute mania occurring as part of bipolar disorders[8].
9	Melatonin	ME	An endogenous hormone, used to regulate sleep-wake cycles and available as an over-the-counter supplement[9].
10	Mirtazapine	MI	A tetracyclic antidepressant used in the treatment of major depression and is used off-label as a drug for insomnia and to increase appetite[10].
11	Tianeptine	TI	An AMPA modulator, is an atypical tricyclic antidepressant with antidepressant and anxiolytic effects primarily used to treat major depressive[11].
12	Lamotrigine	LA	An anticonvulsant, is a phenyltriazine antiepileptic used to treat some types of epilepsy and bipolar I disorder[12].
13	Quetiapine	QU	An antipsychotic, is a psychotropic agent used for the management of bipolar disorder, schizophrenia, and major depressive disorder[13].

14	Chantix	VA	An ACh modulator, is a partial agonist at nicotinic acetylcholine receptors used as an aid in smoking cessation[14].
15	Dextromethorphan	DE	A NMDA receptor antagonist used to treat cases of dry cough[15].
16	Eletriptan	EL	An antimigraine, is a triptan used for the treatment of migraines[16].

*See drug functions at https://go.drugbank.com/ and representative references which are listed in Supplementary References.

Table S2. List of drugs abbreviation and function of Set B

No.	Drug name	Abbr.	Function*
1	γ -aminobutyric acid	AA	An inhibitory neurotransmitter with sedative, hypnotic, anticonvulsant, and hypotensive physiological effects, have also been shown to decrease blood pressure and slow heart rate[17-19].
2	Amitriptyline	AM	A tricyclic antidepressant, for the relief of chronic pain, has been shown to improve cardiac output[20, 21].
3	Aspirin	AS	A salicylate used to treat pain, fever, inflammation, migraines, and reducing the risk of major adverse cardiovascular events[22, 23].
4	Disulfiram	DI	A carbamate derivative used to treat alcohol addiction[24].
5	Dopamine	DA	A catecholamine neurotransmitter used to treat hemodynamic imbalances, poor perfusion of vital organs, low cardiac output, and hypotension[25].
6	Norepinephrine	NE	A sympathomimetic used in the control of blood pressure during various hypotensive states and as an adjunct treatment during cardiac arrest[26, 27].
7	Phenacetin	PH	A painkiller, increases overall rates of cardiovascular disease and hypertension[28].
8	Phenytoin	PT	An anticonvulsant drug used in the treatment of epilepsy and has shown to induce heart block[29].
9	Y-27632 dihydrochloride	Y2	An inhibitor of Rho-associated protein kinase, which has been shown to protect the heart functions[30].

*See drug functions at https://go.drugbank.com/ and representative references which are listed in Supplementary References.

Table S3. Details of cardiac signal processing in MATLAB

1) High-pass filter

The low frequency (<0.2Hz) noise was filtered by a Highpass Butterworth Filter using the "butter" function in MATLAB. The sample code was described below.

```
% Import the signal data
data = xlsread(' Save path of the signal.xlsx');
% Plot the signal pattern
plot = (data);
% Set the cutoff frequency at 0.2 Hz
fc = 0.2;
% Set the sampling frequency at 10 Hz
fs = 10;
% Design a 10th-order Highpass Butterworth Filter
[b,a] = butter(10, fc/(fs/2),'high');
% Signal passing through the highpass filter
fi = filtfilt(b,a,data);
% Plot the filtered signal pattern
figure, plot = (fi);
```

2) Fast Fourier transform (FFT) of the filtered signal

FFT was performed using the "fft" function in MATLAB. The sample code was demonstrated below.

```
% Import the signal data
data = xlsread(' Save path of the signal.xlsx');
% Highpass filter
fc = 0.2;
fs = 10;
[b,a] = butter(10, fc/(fs/2),'high');
fi = filtfilt(b,a,data);
% Sampling frequency
Fs = 10;
% Length of signal
N = 100;
%Compute the FFT of the filtered signal
Y = fft(fi);
%Compute the two-sided spectrum P2.
P2 = abs(Y/ N);
%Compute the single-sided spectrum P1.
P1 = P2(1: N /2+1);
% Compute the frequency domain.
f = Fs*(0:( N /2))/ N;
%Plot the single-sided amplitude spectrum of the filtered signal.
figure,plot(f,P1)
```

3) Extraction of the peaks

The peaks of the signal were extracted by detecting peak-to-peak values. The sample code was described below.

```
% Import the signal data
data = xlsread(' Save path of the signal.xlsx');
% Normalization
Min=min(data);
Max=max(data);
a2=( data ()-Min)/(Max-Min);
% Extract the peaks
c=findpeaks(a2);
% Obtain the location of the local minimum
IndMin=find(diff(sign(diff(a2)))>0)+1;
% Obtain the location of the local maximum
IndMax=find(diff(sign(diff(a2)))<0)+1;
figure; hold on; box on;
% Plot the signal pattern
plot(1:length(a2),a2);
% Mark the position of troughs with black asterisks
plot(IndMin,a2(IndMin),'k*')
% Mark the position of peaks with red asterisks
plot(IndMax,a2(IndMax),'r*')
% Compute the peak-to-peak values
pk2pk=a2(IndMax)-a2(IndMin);
% Plot peaks distribution histogram
figure, h=histogram(pk2pk);
```

Supplementary Movies

Movie S1. Schematic illustration of the work flow in the FC system.

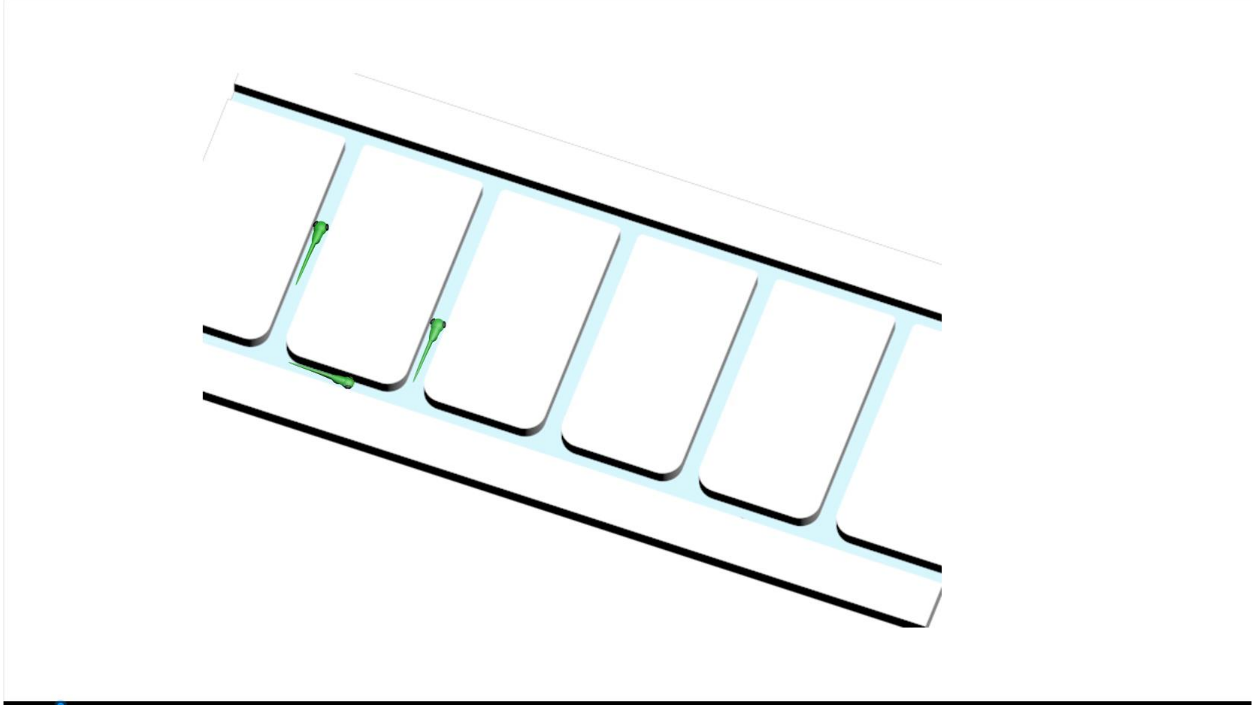

Movie S2. Demonstration of larvae loading, trapping and orientating using a microfluidic chip based on hydrodynamic force.

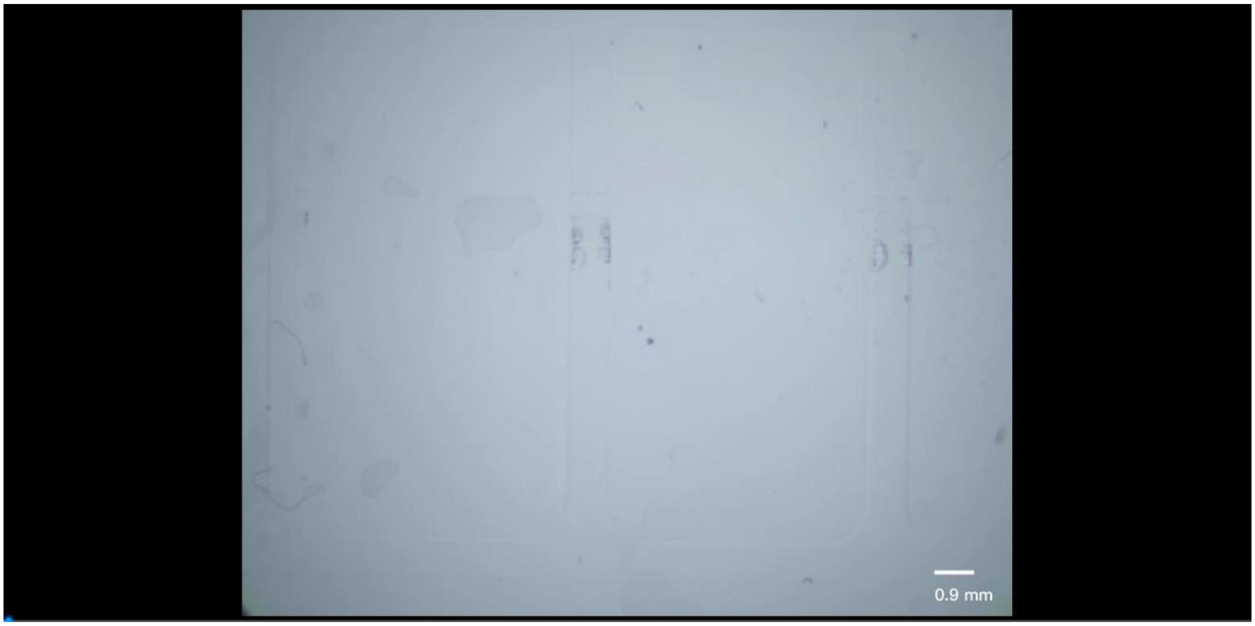

Movie S3. High-throughput microscopic imaging of the animals using the FC system.

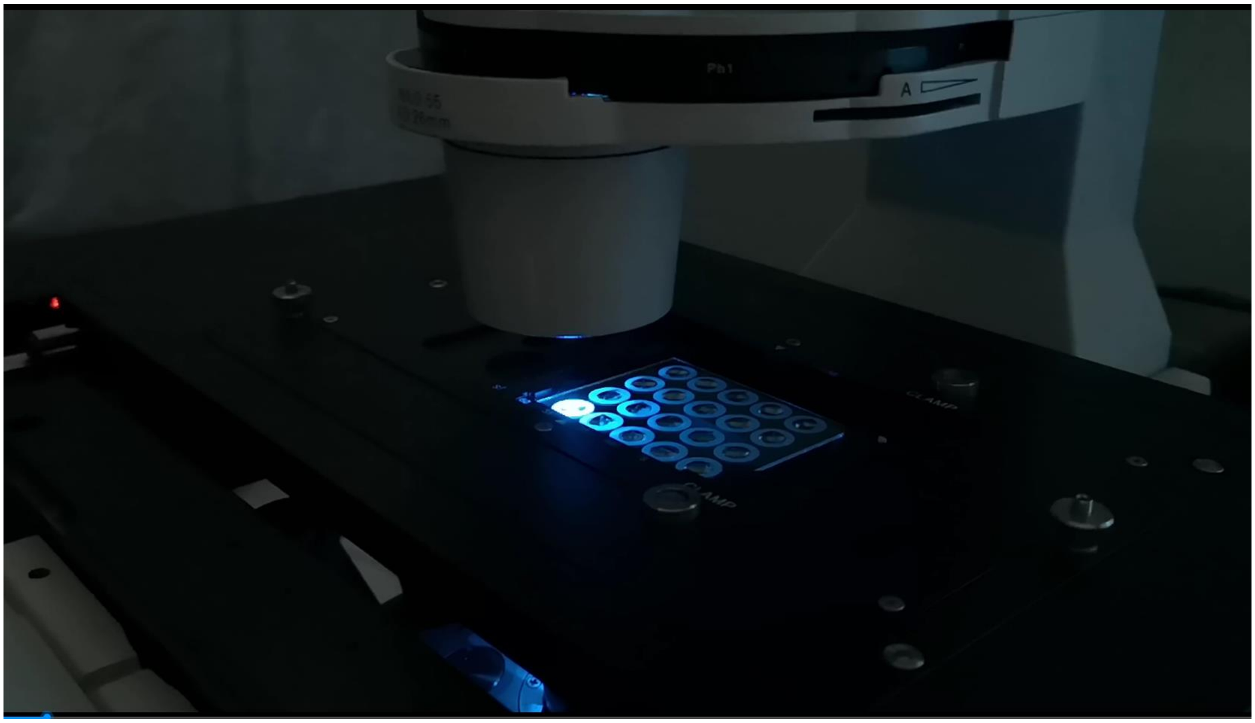

Movie S4. Parallel compound addition using sandwiching method

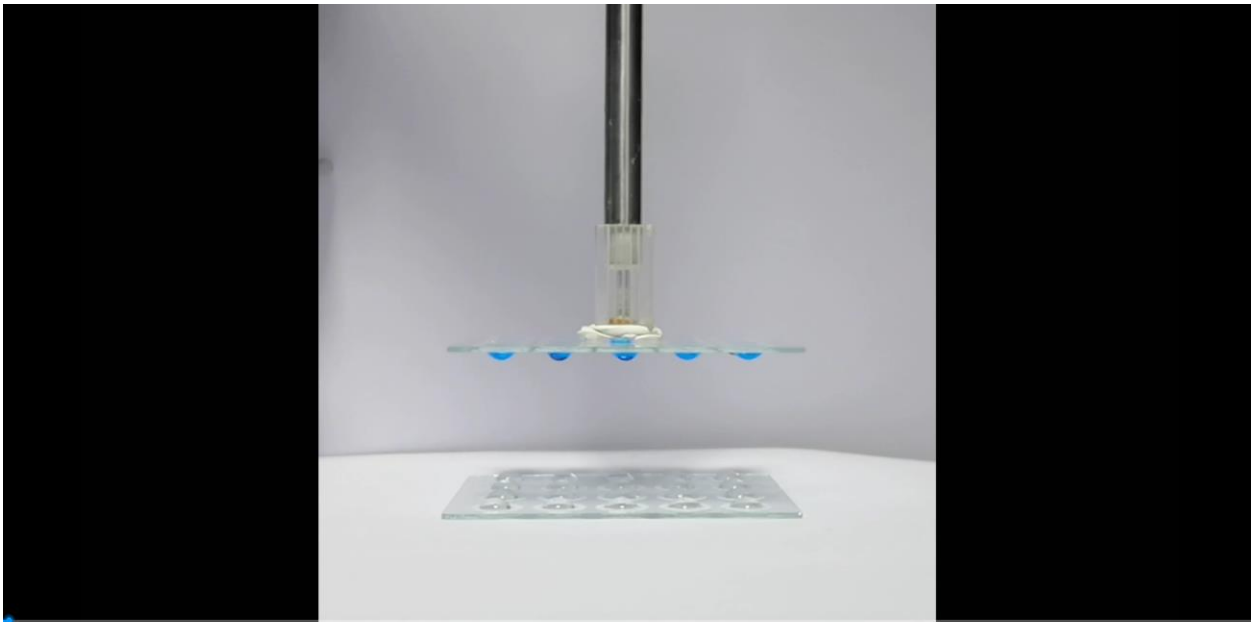

Movie S5: Cardiac recording of the wild-type zebrafish in the capsule (objective: 20x).

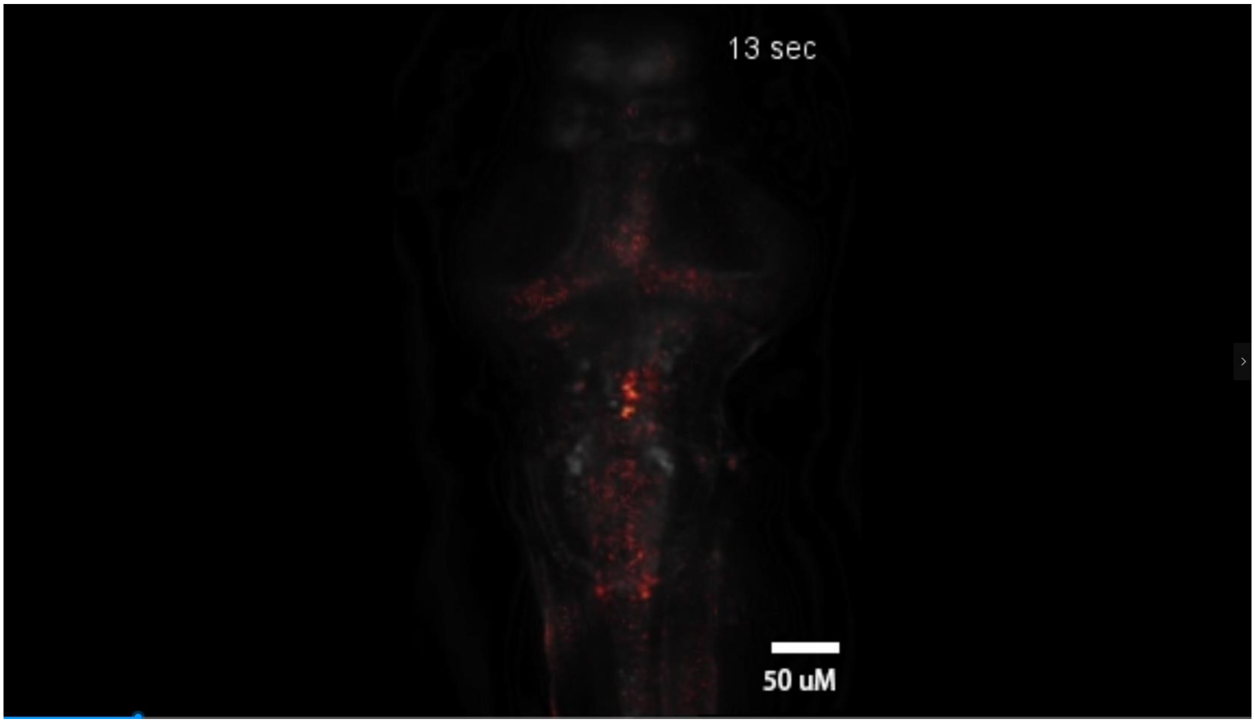

Movie S6. Brain-wide neural activities recording using the FC system (objective: 10x).

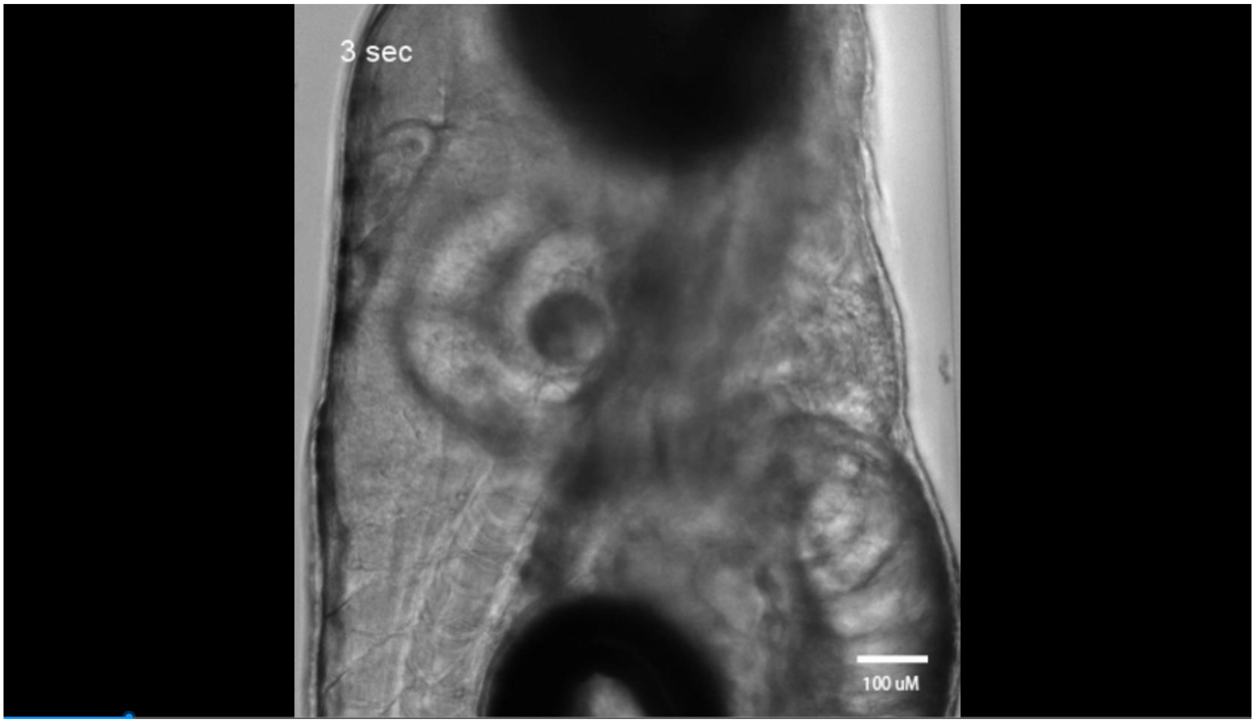

Movie S7: Fluorescent microimaging of the blood cells in the larvae (objective: 5x).

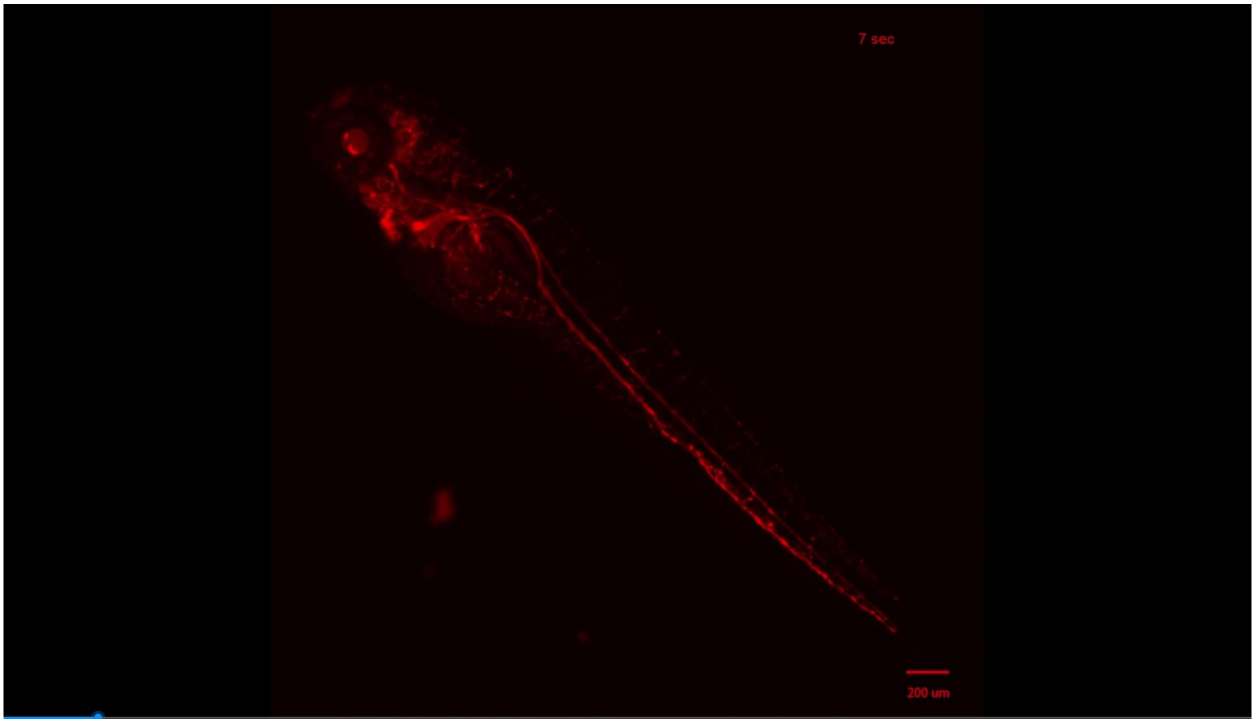

Supplementary References

1. Zhang, F. et al. Clozapine Induced Developmental and Cardiac Toxicity on Zebrafish Embryos by Elevating Oxidative Stress. *Cardiovasc. Toxicol.* **21**, 399-409 (2021).
2. Viana, J. et al. Clozapine-induced transcriptional changes in the zebrafish brain. *NPJ Schizophr.* **6**, 3 (2020).
3. Molly M. Shores , L., Marcella Pascualy , David Flatness , Nancy L. , Richard C. Veith Short-term sertraline treatment suppresses sympathetic nervous system activity in healthy human subjects. *Psychoneuroendocrinology* **26**, 433-439 (2001).
4. Wong, D.T., Perry, K.W. & Bymaster, F.P. Case history: the discovery of fluoxetine hydrochloride (Prozac). *Nat. Rev. Drug Discov.* **4**, 764-774 (2005).
5. Michael E. Thase, M.D., Madhukar H. Trivedi, M.D., and A. John Rush, M.D. MAOis in the Contemporary Treatment of Depression. *Neuropsychopharmacology* **12**, 185-219 (1995).
6. Arain, A.M. & Abou-Khalil, B.W. Management of new-onset epilepsy in the elderly. *Nat. Rev. Neurol.* **5**, 363-371 (2009).
7. Bialer, M. & White, H.S. Key factors in the discovery and development of new antiepileptic drugs. *Nat. Rev. Drug Discov.* **9**, 68-82 (2010).
8. P Seeman, T.T. Antipsychotic drugs which elicit little or no Parkinsonism bind more loosely than dopamine to brain D2 receptors, yet occupy high levels of these receptors. *Mol. Psychiatry* **3**, 123-134 (1998).
9. Melhuish Beaupre, L.M., Brown, G.M., Goncalves, V.F. & Kennedy, J.L. Melatonin's neuroprotective role in mitochondria and its potential as a biomarker in aging, cognition and psychiatric disorders. *Transl. Psychiatry.* **11**, 339 (2021).
10. Gold, S.M. et al. Comorbid depression in medical diseases. *Nat. Rev. Dis. Primers.* **6**, 69 (2020).
11. Butcher, E.C. Can cell systems biology rescue drug discovery? *Nat. Rev. Drug Discov.* **4**, 461-467 (2005).
12. Romoli, M., Sen, A., Parnetti, L., Calabresi, P. & Costa, C. Amyloid-beta: a potential link between epilepsy and cognitive decline. *Nat. Rev. Neurol.* (2021).
13. De Hert, M., Detraux, J., van Winkel, R., Yu, W. & Correll, C.U. Metabolic and cardiovascular adverse effects associated with antipsychotic drugs. *Nat. Rev. Endocrinol.* **8**, 114-126 (2011).
14. Dunlop, J., Bowlby, M., Peri, R., Vasilyev, D. & Arias, R. High-throughput electrophysiology: an emerging paradigm for ion-channel screening and physiology. *Nat. Rev. Drug Discov.* **7**, 358-368 (2008).
15. Murrough, J.W., Abdallah, C.G. & Mathew, S.J. Targeting glutamate signalling in depression: progress and prospects. *Nat. Rev. Drug Discov.* **16**, 472-486 (2017).
16. Silberstein, S.D. Treatment recommendations for migraine. *Nat. Clin. Pract. Neurol.* **4**, 482-489 (2008).
17. Duman, R.S., Sanacora, G. & Krystal, J.H. Altered Connectivity in Depression: GABA and Glutamate Neurotransmitter Deficits and Reversal by Novel Treatments. *Neuron* **102**, 75-90 (2019).
18. Pengju Ma, T.L., Fanceng Ji, Haibo Wang , Juntao Pang Effect of GABA on blood pressure and blood dynamics of anesthetic rats. *Int. J. Clin. Exp. Med.* **8**, 14296-14302 (2015).
19. Schur, R.R. et al. Brain GABA levels across psychiatric disorders: A systematic literature review and meta-analysis of (1) H-MRS studies. *Hum. Brain Mapp.* **37**, 3337-3352 (2016).

20. Zhang, L. et al. Amitriptyline Reduces Sepsis-Induced Brain Damage Through TrkA Signaling Pathway. *J. Mol. Neurosci.* **70**, 2049-2057 (2020).
21. Kim, Y. et al. Amitriptyline inhibits the MAPK/ERK and CREB pathways and proinflammatory cytokines through A3AR activation in rat neuropathic pain models. *Korean J. Anesthesiol.* **72**, 60-67 (2019).
22. Schror, K. & Rauch, B.H. Aspirin and lipid mediators in the cardiovascular system. *Prostaglandins Other Lipid Mediat.* **121**, 17-23 (2015).
23. Chandra, S., Jana, M. & Pahan, K. Aspirin Induces Lysosomal Biogenesis and Attenuates Amyloid Plaque Pathology in a Mouse Model of Alzheimer's Disease via PPARalpha. *J Neurosci.* **38**, 6682-6699 (2018).
24. Munzel, T. & Daiber, A. The potential of aldehyde dehydrogenase 2 as a therapeutic target in cardiovascular disease. *Expert Opin. Ther. Targets* **22**, 217-231 (2018).
25. Bucolo, C., Leggio, G.M., Drago, F. & Salomone, S. Dopamine outside the brain: The eye, cardiovascular system and endocrine pancreas. *Pharmacol. Ther.* **203**, 107392 (2019).
26. Ghasemi, M. & Mehranfard, N. Mechanisms underlying anticonvulsant and proconvulsant actions of norepinephrine. *Neuropharmacology* **137**, 297-308 (2018).
27. Giorgi, F.S., Pizzanelli, C., Biagioni, F., Murri, L. & Fornai, F. The role of norepinephrine in epilepsy: from the bench to the bedside. *Neurosci. Biobehav. Rev.* **28**, 507-524 (2004).
28. Ramos, B.P. & Arnsten, A.F. Adrenergic pharmacology and cognition: focus on the prefrontal cortex. *Pharmacol. Ther.* **113**, 523-536 (2007).
29. Guldiken, B., Remi, J. & Noachtar, S. Cardiovascular adverse effects of phenytoin. *J. Neurol.* **263**, 861-870 (2016).
30. Cadete, V.J. et al. Effect of the Rho kinase inhibitor Y-27632 on the proteome of hearts with ischemia-reperfusion injury. *Proteomics* **10**, 4377-4385 (2010).
